# Latent Variables Quantifying Neighborhood Characteristics and Their Associations with Poor Mental Health

**DOI:** 10.3390/ijerph18031202

**Published:** 2021-01-29

**Authors:** Katherine L. Forthman, Janna M. Colaizzi, Hung-wen Yeh, Rayus Kuplicki, Martin P. Paulus

**Affiliations:** 1 Laureate Institute for Brain Research, 6655 South Yale Avenue, Tulsa, OK 74136, USA; kforthman@laureateinstitute.org (K.L.F.); jcolaizzi@laureateinstitute.org (J.M.C.); hyeh@cmh.edu (H.-w.Y.); rkuplicki@laureateinstitute.org (R.K.); 2 Department of Mathematics, College of Engineering & Natural Sciences, The University of Tulsa, 800 South Tucker Drive, Tulsa, OK 74104, USA; 3 Division of Health Services and Outcomes Research, Children’s Mercy Hospital, 2401 Gillham Road, Kansas City, MO 64108, USA

**Keywords:** neighborhood, factor analysis, mental health

## Abstract

Neighborhood characteristics can have profound impacts on resident mental health, but the wide variability in methodologies used across studies makes it difficult to reach a consensus as to the implications of these impacts. The aim of this study was to simplify the assessment of neighborhood influence on mental health. We used a factor analysis approach to reduce the multi-dimensional assessment of a neighborhood using census tracts and demographic data available from the American Community Survey (ACS). Multivariate quantitative characterization of the neighborhood was derived by performing a factor analysis on the 2011–2015 ACS data. The utility of the latent variables was examined by determining the association of these factors with poor mental health measures from the 500 Cities Project 2014–2015 data (2017 release). A five-factor model provided the best fit for the data. Each factor represents a complex multi-dimensional construct. However, based on heuristics and for simplicity we refer to them as (1) Affluence, (2) Singletons in Tract, (3) African Americans in Tract, (4) Seniors in Tract, and (5) Hispanics or Latinos in Tract. African Americans in Tract (with loadings showing larger numbers of people who are black, single moms, and unemployed along with fewer people who are white) and Affluence (with loadings showing higher income, education, and home value) were strongly associated with poor mental health ($R^{2}=0.67$, $R^{2}=0.83$). These findings demonstrate the utility of this factor model for future research focused on the relationship between neighborhood characteristics and resident mental health.

## 1. Introduction

How we characterize neighborhoods has implications for identifying ways in which the neighborhood environment affects the health of its residents at both the individual and population level. The research in this area has generally centered around the characteristics of a neighborhood such as socioeconomic composition, housing quality, walkability, transportation options, social cohesion, or safety that may have implications for (1) community development and resident access to resources [1] and (2) resident mental and physical health outcomes [2]. Regarding health outcomes, some of the most consistent findings documented in previous studies and systematic reviews include reports that, while accounting for individual-level factors, neighborhood characteristics are associated with mortality rates [3], early childhood health outcomes [4], general mental health [5], and depression [6]. Though a link has been established, there is a lack of an approach for characterizing a neighborhood that (1) is widely-used and objective, (2) uses publicly-available, regularly updated data, (3) uses a large, nation-wide sample, and (4) is low in dimension without sacrificing information. As a result, there is no clear consensus regarding the strength of the relationship neighborhood characteristics have with resident health and the features of the neighborhood environment that are related to that effect. Objectively delineating the characteristics of a neighborhood and evaluating their relationship to mental health could have important implications for future research linking social factors to the biological processes underlying psychiatric disorders. Examining this first on a nationwide level would allow researchers to ascertain a broad scope of the characteristics that are prevalent across cities before further outlining their effects on the residents. Below, we present a systematic approach to addressing this methodological gap by using factor analysis to delineate characteristics of a neighborhood on a nation-wide level. We then demonstrate the utility of this new model by examining the association between these neighborhood factors and mental health outcomes across multiple cities and the applicability of these methods for use in future research.

### 1.1. Background

The characteristics of a neighborhood have long been recognized to contribute to the well-being of its residents such that individuals in more disadvantaged neighborhoods often report higher levels of physical and mental health symptoms [7,8,9]. Several neighborhood characteristics have been linked to mental health outcomes, particularly depression [6]. A majority of this research revolves around the often interdependent constructs of neighborhood socioeconomic status (NSES; most frequently measured as neighborhood-level income, education, and occupation) [10] and neighborhood built environments (access to parks, quality of housing, location and maintenance of local roads, etc.) as impacting resident mental health [11]. For instance, various neighborhood characteristics including socioeconomic composition [9], safety [12], quality [11], social cohesion [13], and resource availability (e.g., access to health care, transportation, or green space) [14,15] have been significantly related to depressive symptoms.

The methodologies across many of these studies, however, are inconsistent in ascertaining or selecting a subset of a population, extracting neighborhood characteristics and physical boundaries, or focusing on specific indices [6]. For instance, a 2008 review by Mair et al. identified 45 studies, mostly cross-sectional, relating neighborhood characteristics and mental health, in particular, depression [6]. Their review presents studies with varying neighborhood definitions (e.g., boundary definitions ranging from participant-defined borders to various measures from census data), study populations (e.g., variations in age, race/ethnicity), measurement of neighborhood characteristics (e.g., socioeconomic status, racial/ethnic composition, mobility, built environment, urbanicity, disorder/crime/violence, resident social interactions), and analytic approaches (e.g., linear single- and multi-level models, structural equation modeling). The large variability in these studies makes it difficult to draw consistent conclusions on the topic and Mair et al. ultimately conclude that reducing the variability in measurement of neighborhood characteristics is needed to empirically test theories on this relationship. Likewise, a 2015 review by Oakes et al. identified 1369 papers reporting on social epidemiological neighborhood effects on resident physical and mental health outcomes as well as social behavior (e.g., criminal behavior) [16]. Both experimental and observational designs were included in the review, and of all the relevant papers, only a handful described an impact of neighborhood on health, leaving Oakes et al. to conclude that the scientific evidence for neighborhood effects remains inconsistent. As emphasized in these reviews, the lack of consensus in measurement, definitions, and techniques makes it difficult to determine to what degree any reported neighborhood influences are due to the specific definition or share a common process that contributes to poor mental health.

Other challenges to consistency and interpretability come in the use of subjective measurements and limited variable selection. For instance, various studies have used researcher observations and resident interviews to define both the physical boundaries of a neighborhood (e.g., [17]) as well as characteristics of the neighborhood and its residents (e.g., [18]). Such methods provide many variables of interest, including resident perception of the safety or quality of a neighborhood, and allow researchers to decompose analyses from aggregate data to specific and individual contexts. However, the use of uniform classifications of neighborhood physical boundaries via census tracts rather than participant- or researcher-defined areas would afford such analyses additional consistency, reproducibility, and objectivity. Furthermore, amidst the current literature, many studies only evaluate the effects of one neighborhood characteristic—commonly NSES or racial composition—rather than looking at a broader range of variables describing the neighborhood. Without the use of a systematic approach to classifying neighborhood characteristics, studies are limited in their ability to address a range of variables in part due to the multicollinear nature of neighborhood characteristics. Therefore, analyses addressing these associations would benefit from a uniform, objective, and reproducible method of defining neighborhood boundaries (such as those defined by census tracts) and characteristics (such as those accounting for multiple variables like factor analysis).

In light of this need for more uniform and reproducible methodological techniques to classifications of neighborhood effects, a few studies have presented promising approaches. Kolak et al. [19], for instance, used an unsupervised machine learning approach to reduce the dimensionality of multivariate neighborhood data extracted from the U.S. Census. Their goal was to predict mortality rates in a specific geographical area based on social determinants of health including advantage, isolation, opportunity, racial cohesion, and so forth. Similarly, Galster et al. [1] described a method that used neighborhood characteristics to monitor and predict demographic, economic, and housing changes over time. In part, Galster et al. used exploratory factor analysis with census tracts and neighborhood data from various publicly-available data sets within five U.S. cities over the course of two consecutive two-year periods. The methods of Kolak et al. and Galster et al. offer empirical ways to broaden the scope of neighborhood-level analyses from a single NSES or deprivation index to the consideration of multivariate factors contributing to resident health and neighborhood outcomes. However, both the aforementioned analyses were confined to limited geographical regions within the U.S. which reduces sample variability and the generalizability of their findings. A study by Miles et al. describes a method for measuring NSES using factor analysis and the American Community Survey (ACS) that can be explored longitudinally [20]. Their method aimed to find significant neighborhood characteristics based on the time-invariance of a factor describing NSES. The statistical approach of Miles et al. is promising, however, it is still limited in its range of neighborhood characteristics addressed and the demonstration of any potential predictive abilities of these characteristics. Finally, a study by Li et al. combined factor analysis and cluster analysis in a multivariate-structural approach to to assess the effect of neighborhoods on an individual’s health [21]. However, Li et al. (1) analyzed data from outside the U.S. providing limited applicability to a U.S. population and (2) excluded income and minority population, two variables that have been reported to be integral to both neighborhood characterizations and resident health outcomes [22]. Thus, the wide variability in research techniques complicates the ability to draw clear conclusions and adequately address the mental health needs within these populations. Furthermore, it suggests that a systematic, reproducible, and comprehensive approach on a nation-wide level has been lacking and could provide a crucial step forward to identify and address specific environmental factors influencing rates of poor mental health.

### 1.2. Current Study

The primary aim of this paper is to address some of the methodological gaps presented in recent reviews [2,6,16]. We aim to do this by (1) building on previous latent-variable approaches described by Miles et al. and Li et al. by comprehensively quantifying neighborhood characteristics using a wide range of demographic variables sampled across the U.S. and (2) examining the associations of the identified factors with community mental health outcomes in order to demonstrate the applicability of this approach. To achieve this, we utilized data provided by (1) the ACS, a household survey conducted by the U.S. Census that covers a variety of demographic statistics and includes a highly representative sample of the U.S. population, and (2) the 500 Cities Project, which provides neighborhood-level mental health data including prevalence rates for the 500 largest cities in the U.S. [23,24], making these ideal data sets for studying population-level demographics and associated mental health.

## 2. Materials and Methods

### 2.1. Statistics for Neighborhood Characteristics

The ACS, inaugurated in 2005, uses continuous measurement methods based on a series of monthly samples to produce annual population estimates for small areas. Each year, the ACS surveys approximately 3.54 million addresses across the country. This sample is sufficient for the Census to provide reliable 5-year estimates for geographical areas called census tracts. Census tracts are areas primarily defined by population density and have a population of between 1200 and 8000. For the current study, neighborhood data were obtained at tract-level from the 5-year estimate spanning 2011–2015 [25].

The ACS language assistance program utilizes methods and procedures that assist sample households with limited English proficiency in order to reduce the chance of nonresponse bias [26].

The ACS samples both home addresses and group quarters (places where a group of people live together, such as nursing homes, college dormitories, and homeless shelters). Approximately 2.5% of the expected population inhabiting group quarters was sampled [26].

### 2.2. Neighborhood Mental Health Statistics

Mental health data were obtained from the 500 Cities Project, which provides tract-level mental health data for 27,204 tracts within the largest 500 cities in the United States. The 500 Cities Project uses Small Area Estimation (SAE) to estimate statistics from the Behavioral Risk Factor Surveillance System Survey (BRFSS) data at the census-tract level. The BRFSS is conducted by a telephone survey interviewing approximately 400,000 adults across the United States and its territories [23], and is administered continuously through the year [27]. The survey is available in both English and Spanish, and states have the option of translating the questionnaires into additional languages [28]. Response rates are reported in the BRFSS 2014 and 2015 Summary Data Quality Reports [29,30]. The BRFSS uses iterative proportional fitting to weigh statistics by age, gender, race and ethnicity, and geographical region [27]. The Centers for Disease Control and Prevention (CDC) derives individual mental health status from the following question in the BRFSS: *“Now thinking about your mental health, which includes stress, depression, and problems with emotions, for how many days during the past 30 days was your mental health not good?”*. The 500-Cities project then aggregates the individual-level data from this question into tract-level SAEs of the proportion of individuals ≥ 18 years old within a tract who responded that they had ≥14 bad mental health days within the last 30 days. For the current study, mental health data was obtained at the tract-level from the 500 Cities 2017 release, containing SAE estimates of the 2014–2015 BRFSS data [24].

The BRFSS was initially designed to produce state-level estimates [31]. Therefore, unsurprisingly, the sample of 400,000 adults in the BRFSS is insufficient to provide estimates at the census-tract level. SAE allows for the derivation of statistics for under-sampled small geographic areas, such as census tracts. The mental health variable we used to explore the relationship between the neighborhood factors and mental health is a SAE derived by the CDC 500 Cities Project using a Multilevel Regression with Poststratification (MRP) approach. The SAE was constructed in 4 steps. First, a multilevel logistic model was fitted to data from the BRFSS survey. The multilevel model was then applied to the census population, and then model-based SAEs were generated via poststratification using ACS variables. Finally, the SAEs were validated by comparison to BRFSS direct estimates for all 50 states and DC and for all counties with at least 50 respondents. The ACS measures included in the poststratification were age, sex, race/ethnicity, and county-level poverty. More details on the derivation of the SAE can be found at [32,33].

### 2.3. Latent Variable Extraction for Neighborhood Characteristics

In order to use as many of the statistics provided by the ACS as possible, we used a latent variable approach to arrive at a multivariate quantitative characterization of the neighborhood. This method gave us the opportunity to objectively select variables to include in our analysis based on their contribution to the variance between neighborhoods. We assumed that these statistics are linked by underlying, latent variables which allowed us to work with a smaller number of variables without sacrificing much information. We applied a factor analysis method after normalizing the variables as described in Section 2.3.1.

The current study aimed to use data at the smallest geographic division possible for a fine-grained view of living environment. The two smallest geographic divisions available in the ACS data set are block-groups and tracts. On average, a block group is 1/3 the size of a tract. We performed the factor analysis on both levels and the resulting factors were largely the same (see Comparison to Block Group, Supplementary Methods, and Figure S1). Though larger, the tract level factors were chosen because (1) there were variables of interest that were not available at the block group level, namely disability status, citizenship status, and mobility, (2) the tract-level statistics have a smaller margin of error, and (3) the 500 Cities Project data are only available at the tract level.

#### 2.3.1. Feature Selection

We included 37 ACS data tables in the analyses which contained 461 measures describing tract characteristics of age, race/ethnicity, citizenship, nativity, mobility, means of transportation to work, household type, marriage status, education level, disability status, income, employment status, home type, housing cost, and residential tenure (Tables S1–S37). Among these 461 measures, 215 were removed due to redundancy or low variability. We defined redundancy as variables that were sufficiently represented by another variable (for example, the female population is redundant because it is the inverse of the male population). We defined low variability as a coefficient of variation lower than 0.06 across tracts. Data reduction details are provided in a flow chart in Figure S2. We combined the remaining 246 statistics describing strata or subgroups (e.g., age groups, gender, education levels) to form single statistics. This selection process led to a final number of 39 measures for subsequent analyses. For further details, see Feature Selection, Supplementary Methods.

We subjected these measures to a heuristic, data-driven transformation approach to approximate Gaussian distributions as close as possible (see Transformation, Supplementary Methods). We imputed missing values (0.16% of the dataset) using the weighted average of 10-nearest neighbors after transformation.

#### 2.3.2. Factor Analysis

We entered a total of 39 transformed and/or imputed measures into an exploratory factor analysis to investigate the underlying latent variable structure. Factor loadings were estimated by the minimum residual method, and oblimin rotation was applied to improve interpretation. We explored a range between 1 and 12 factors and chose the factor number based on Kaiser’s rule (i.e., keeping factors with eigenvalues at least 1), a scree plot, the amount of total variance explained from each model produced, and the interpretability of the factor structure. The stability of the final factor model was examined by 2000 bootstrapped samples and the standard error was calculated for each loading of each variable within each factor. The factor scores were computed for all U.S. tracts. Because oblique rotation leaves the factors correlated, we also examined the correlation between each of the factors using Pearson correlation.

### 2.4. Relationship between Neighborhood Latent Variables and Mental Health

In order to explore how the factors are associated with poor mental health rates, we examined the relationship between the latent variables and the 500 Cities project measure of poor mental health rates, which is quantified as the proportion of individuals in a neighborhood reporting at least 14 participant-defined “bad mental health days” within the past 30 days.

We merged the factor scores for each census tract with data from the 500 Cities Project and investigated their associations by bivariate and multivariable approaches. A comparison of the ACS and BRFSS sample characteristics is provided in Table S39.

#### 2.4.1. Bivariate Associations

We first described the bivariate associations by smoothing splines. We fit a separate spline for each state to account for regional differences in the relationship. We summarized the fit of the splines using median and inter-quartile range (IQR) of the $R^{2}$ statistic across states.

#### 2.4.2. Multivariable Approach

The derivation of the SAE using ACS variables leaves a possibility that sex, age, race/ethnicity, and income measures may confound the relationship between the mental health statistic and the neighborhood factors. To address this issue, we used a multivariable approach described by Selya et al. [34] to derive Cohen’s $f^{2}$ statistic and the unique variance explained by each neighborhood factor. The $f^{2}$ statistic measures local effect size within a linear mixed effects model (LME). Each of the following LMEs described here share the 500 Cities Project tract-level small area estimate of poor mental health rate as the dependent variable. We created an LME model for each factor, denoted $M_{a,i}$, that included fixed effects of the ACS variables describing sex, age, race, ethnicity, and poverty status, the ith neighborhood factor, and random state and county intercepts. Each model $M_{a,i}$ was then compared to an LME model, denoted $M_{a}$, that included all the random and fixed effects in $M_{a,i}$ except the neighborhood factor. The null model, $M_{null}$, which included only the random effects, was used to compute $R^{2}$ of each model, and was defined as

$$R_{model}^{2}=\frac{V_{null}-V_{model}}{V_{null}},$$

where *V* denotes variance. The unique variance explained by each neighborhood factor was calculated as $R_{a,i}^{2}-R_{a}^{2}$. The fixed effect size for the neighborhood factor in the mixed model is estimated by Cohen’s $f^{2}$ statistic:

$$f_{i}^{2}=\frac{R_{a,i}^{2}-R_{a}^{2}}{1-R_{a,i}^{2}}.$$

A final LME model was created that included the ACS variables, all the neighborhood factors, and the random effects, and was denoted $M_{a,1:5}$. The unique variance explained by all the neighborhood factors and the effect sizes were derived from this model.

According to Cohen, as a general rule of thumb, a small effect size is defined as $f^{2}\geq 0.02$, a medium effect size is defined as $f^{2}\geq 0.15$, and a large effect size is defined as $f^{2}\geq 0.35$.

### 2.5. Software

We used the statistical software R [35] for all data extraction, analyses, and the generation of all figures. The R code for this manuscript is available as a supplement [36]. ACS data were obtained through the R package *acs* [37]. The *e1071* [38] and *scales* [39] packages were used for transformation, the *DMwR* [40] package for imputation, and the *psych* package [41] for factor analysis.

## 3. Results

### 3.1. Exploratory Factor Analysis

**Figure 1:**
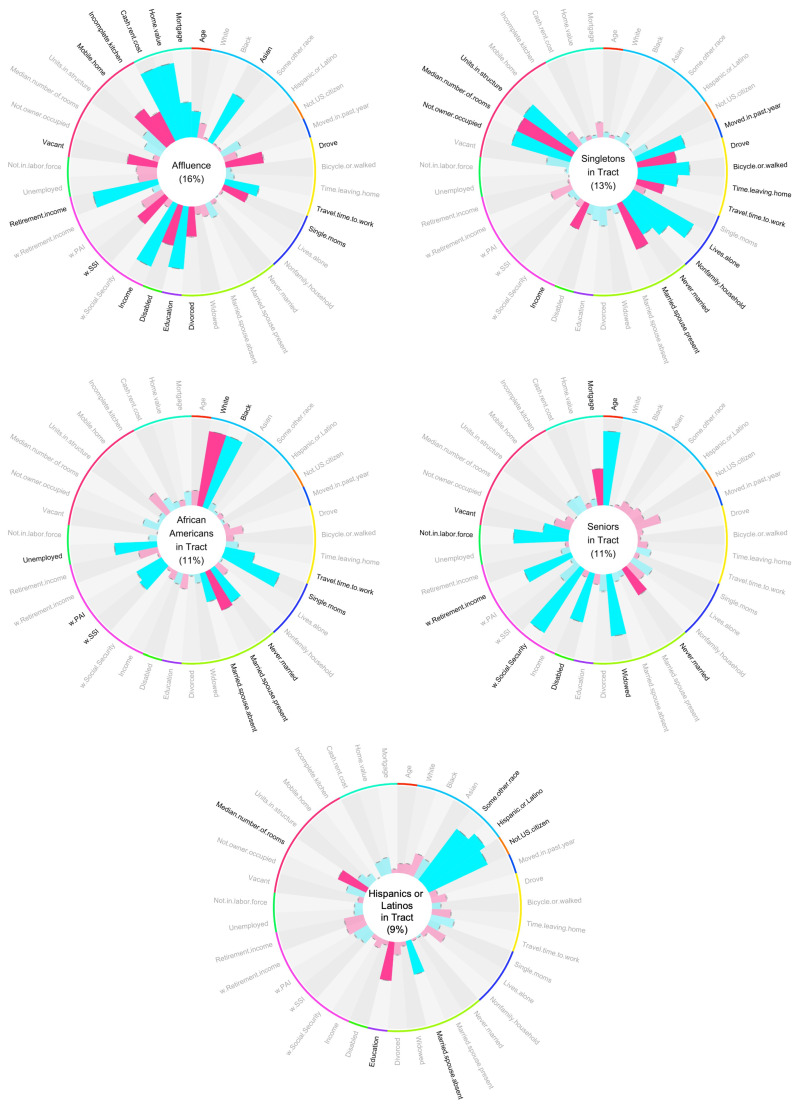
The Factor Structure. Each circular bar plot is a visual representation of a single latent factor. The name of the factor is in the center of the plot. Each bar represents the loading of an input variable to the factor. Blue bars indicate a positive loading, while pink bars indicate a negative loading. Variables with loadings > 0.3 to the factor are bolded. Input variables are grouped by type with the colored lines around the edge of each plot. These groups (starting from the top, moving clockwise) encompass age (red), race and ethnicity (sky blue), nativity and citizenship (orange), mobility (blue), transportation to work (yellow), household type (navy blue), marital status (lime green), education level (purple), disability status (green), income (magenta), employment (mint green), residential conditions (pink), and tenure (turquoise). A larger version of these plots is given in the supplement (Figures S6–S10).

We selected a 5-factor model, depicted in Figure 1. The factor structures of the 4- and 6-factor models are shown in Figures S3 and S4 for comparison purposes. A factor model with up to eight factors had eigenvalues greater than 1.0. The scree plot shows an ‘elbow’ at five factors (Figure S5). The five-factor model accounted for 60% of the variance, reproduced 0.98 of the off-diagonal elements of the sample correlation matrix, and the root-mean square of residuals (RMS) was 0.04. Fit statistics of the 12 factor models explored are given in Table S38. The five factors were labeled after the variables with the strongest absolute loadings into each factor. It is important to note that the factor names are arbitrary labels of unknown latent variables, and factors are named solely for ease of reference. The factor names do not fully encompass what the factor describes, and we do not suggest that the latent variable the factor describes is the same as the factor’s name. We use a simple heuristic to name the factors: if the highest loading variables can all be described using one word, we use that word. Otherwise, we name the factor after the highest loading variable. The factor names chosen were (1) Affluence, (2) Singletons in Tract, (3) African Americans in Tract, (4) Seniors in Tract, and (5) Hispanics or Latinos in Tract (Figure 1). Affluence, which accounted for 16% of the variance, showed greatest loadings from tract statistics relating to NSES, such as income (0.79 for Income in the circle plot) and education (0.73 for Education). Singletons in Tract, which accounted for 13% of the variance, demonstrated strong loadings from the proportion of people living alone (0.81 for Lives.alone), the average number of housing units per structure (0.72 for Units.in.structure), and the proportion of homes in a tract not occupied by their owner (0.70 for Not.owner.occupied). African Americans in Tract, which accounted for 11% of the variance, was positively associated with the proportion of the population who self-identified their race as ‘Black or African American alone’, (0.87 for Black), and inversely associated with those self-identifying as ‘White’ (−0.87 for White). Though the terms ‘Black’ and ‘African American’ are not synonymous, they are grouped together in the U.S. Census. Therefore either term could be used to describe this factor; we arbitrarily chose African American. This factor was also loaded highly on the proportion of single moms (0.69 for Single.moms), lack of married couple family homes (−0.49 for Married.spouse.present, 0.46 for Never.married), the unemployed population (0.49 for Unemployed), and the proportion of people living on government assistance such as Supplemental Security Income (SSI) or Public Assistance Income (PAI) (0.37 for w.SSI, 0.34 for w.PAI). Seniors in Tract, which also accounted for 11% of the variance, was primarily related to age (0.85 for Age) and the proportion of the population receiving Social Security Income (0.87 for w.Social.Security). Hispanics or Latinos in Tract, which accounted for 9% of the variance, was strongly related to the proportion of the population self-identifying their race as a race other than white, black, or Asian (0.74 for Some.other.race) and the population self-identifying their ethnicity as Hispanic or Latino (0.83 for Hispanic.or.Latino), as well as the population of non-U.S. citizens (0.76 for Not.US.citizen).

The oblique rotation procedure left the factors correlated (Figure S11): African Americans in Tract was correlated with Hispanics or Latinos in Tract (r=0.36), Singletons in Tract (r=0.33), and Affluence (r=−0.29); Seniors in Tract was correlated with Hispanics or Latinos in Tract (r=−0.26) and Affluence (r=−0.21). Least correlated were Hispanics or Latinos in Tract and Affluence (r=−0.08).

### 3.2. Associations between Neighborhood Factors and Prevalence of Poor Mental Health

#### Results from the Bivariate Approach

**Figure 2:**
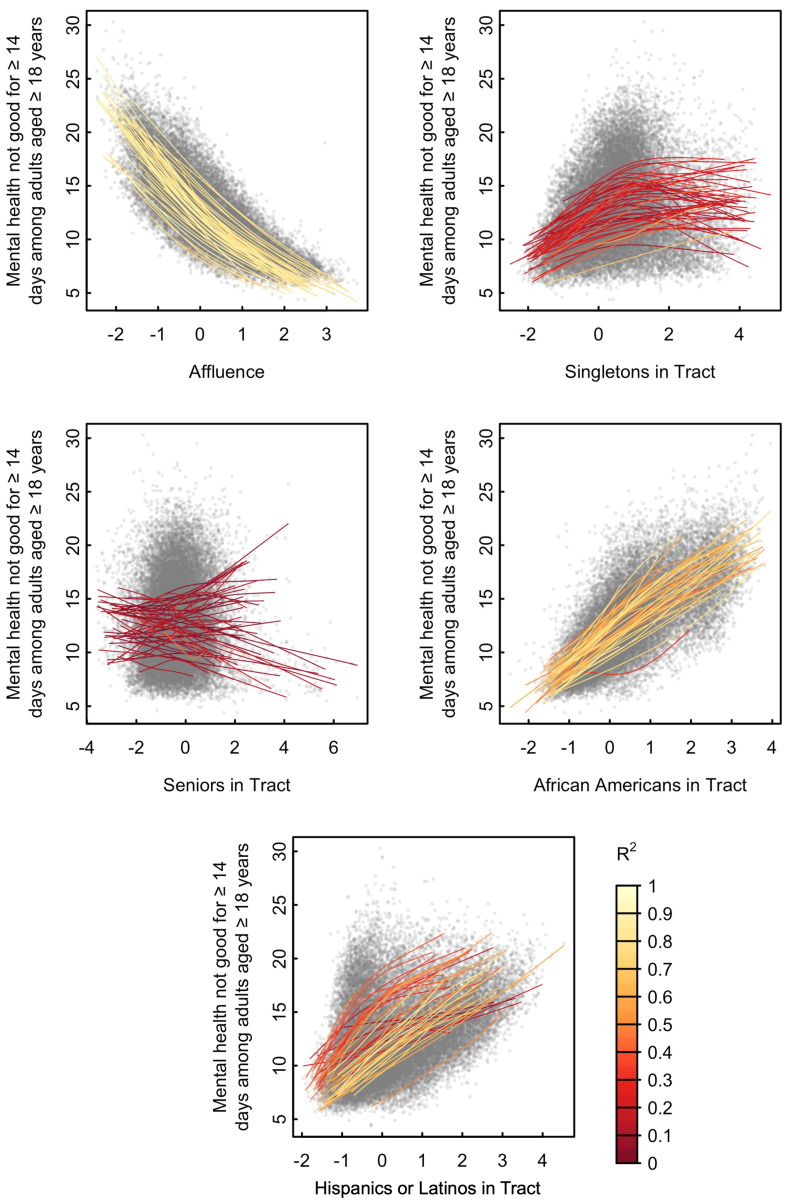
Relationship Between Factors and Mental Health. Proportion of residents over 18 who have experienced ≥14 days of bad mental health during the past 30 days from the 500 Cities Project vs. neighborhood factor scores. Each point on the plot represents a single tract. A separate cubic spline (colored curve) was fit to tracts of each state.

The prevalence of individuals in a tract with 14 or more days of poor mental health appeared to be most related to Affluence, followed by African Americans in Tract, Hispanics or Latinos in Tract, Singletons in Tract, and least related to Seniors in Tract (Figure 2). There was an obvious inverse relationship between the poor mental health measure and Affluence of tracts for all states (median R-square 0.83 and IQR between 0.80 and 0.86, Figure S12). There also existed monotone, increasing trends between the health measure and the two factors African Americans in Tract (median and IQR $R^{2}$: 0.67 (0.58, 0.74)) and Hispanics or Latinos in Tract (median and IQR $R^{2}$: 0.49 (0.35, 0.67)), despite higher variability in trends across states for the latter. Concave trends appeared between the poor mental health outcome and Singletons in Tract for most states (median and IQR $R^{2}$: 0.17 (0.11, 0.24)). The uniform relationship for tracts with a lower Singletons in Tract score indicates that neighborhoods with fewer singletons tend to have lower rates of poor mental health. As the factor score increases, however, rates of mental health become more variable, indicating there is no relationship between mental health and a higher Singletons in tract score. Seniors in Tract showed different patterns across states, with a mixture of positive and negative, linear and concave trends (median and IQR $R^{2}$: 0.05 (0.02, 0.11)).

### 3.3. Results from the Multivariable Approach

Although the unique variance explained by each factor is attenuated by the ACS variables used for the SAE (age, sex, race, ethnicity, and poverty), all factors apart from Singletons in Tract explain unique variance in the mental health variable after subtracting the variance explained by the ACS variables (Table 1). The effect size of Affluence far exceeds a large effect size ($f^{2}\geq 0.35$), demonstrating that Affluence explains substantial unique variance beyond the ACS variables. The effect sizes of Seniors in Tract, African Americans in Tract, and Hispanics or Latinos in Tract are all considered medium effect sizes ($f^{2}\geq 0.15$). The effect sizes of those factors are likely more attenuated than that for Affluence because these factors had strong loadings for either age, race, or ethnicity. Singletons in Tract has an effect size of 0, meaning that there is no unique variance explained by Singletons in Tract beyond the ACS variables. This suggests the possibility that the weak relationship discovered between Singletons in Tract and the mental health variable by the bivariate approach is due to confounding of the ACS variables. These results support the results from Section 3.2, showing that the relationships discovered via cubic splines were not reliant on confounding by the use of the ACS variables in the SAE.

## 4. Discussion

This study aimed to (1) quantify neighborhood characteristics using a latent variable approach performed on census data and (2) determine the utility of these latent variables by relating them to mental health outcomes. There were two main results. First, five factors, Affluence, Singletons in Tract, African Americans in Tract, Seniors in Tract, and Hispanics or Latinos in Tract, have demonstrated to be highly representative by accounting for 60% of the neighborhood tract variance and providing a multidimensional assessment of census tracts. Second, the two factors shown by the bivariate analysis to be most strongly related to tract-level descriptors of poor mental health were Affluence and African Americans in Tract. The multivariable analysis shows that all the factors except Singletons in Tract explain unique variance and have medium or large effect sizes after removing the variance explained by sex, age, race, ethnicity, and poverty. Additionally, the relative strength of some effects change with the multivariate analysis, for example, suggesting that Hispanics or Latinos in Tract has an effect almost twice as strong as African Americans in Tract. The difference in ordering of effect sizes between the bivariate and multivariable results highlights the importance of confounders and the risk of overinterpreting either set of results. Taken together, this study shows that census tracts can be robustly quantified using five dimensions and that some of these latent variables are associated with tract-level mental health status.

### 4.1. Latent Factors of Neighborhood Characteristics

While several studies have described the relationship between neighborhood characteristics and mental health [6,42,43], to our knowledge, no previous study has used a latent variable approach on a data set that (1) is publicly-available and regularly updated, (2) represents a large, nation-wide sample, and (3) includes a large number of statistics to extract a multi-dimensional set of latent variables for the characterization of neighborhoods. Similar approaches have been reported that utilize publicly available, regularly updated census data. Kolak et al. [19] used a similar machine learning dimension reduction method on U.S. Census data and reported similar factors to those described here, including one comprised of mainly sociodemographic variables (i.e., Affluence), primarily older adults (i.e., Seniors in Tract), and primarily renters (i.e., Singletons in Tract), further validating our avenue of analysis. Our approach adds to this method by addressing the association with mental health. Additionally, we expand other similar studies such as Galster et al. [1] by leveraging a nation-wide sample, thus increasing the generalizability of our factors. Finally, a recent study by Hu et al. shows a clear relationship between NSES and health [44], however, the results of those analyses are limited because—as in many other papers—only NSES was examined as a predictor of health while the possible influence of other neighborhood characteristics was ignored. The factor analysis approach we report, however, accounts for a wide variation in neighborhood characteristics reduced to a 5-factor, latent variable structure.

Another important notion to consider is time invariance, or how these factors translate across time. For instance, the Area Deprivation Index (ADI) used in Hu et al. [44], developed by Singh [45], was based on a single-factor analysis using 17 socioeconomic indicators selected by Singh from the 1990 U.S. census. This ADI statistic used in Hu et al. was not tested for time invariance, which consequently inherently assumes the factor structure is consistent over time. This assumption may lead to biased results if time invariance of the factor structure does not hold [46]. An index should be based on data in the relevant time period if time invariance has not been demonstrated. While we do not test for time invariance, a new factor model for the time period of interest can be easily calculated for any 5-year period after 2005 using the methods we describe in this paper.

### 4.2. Utility and Viability of Neighborhood Factors

Our factors (Affluence, African Americans in Tract, Hispanics or Latinos in Tract, Singletons in Tract, and Seniors in Tract), have all been explored to some extent in past research and our findings support previous literature.

The relationship between Affluence and mental health appears to follow an exponential decay, suggesting that as Affluence decreases, it corresponds to a greater incremental change in the prevalence of poor mental health. Affluence appears to be related to such measures as socioeconomic status, economic disadvantage, and neighborhood deprivation as described in several previous studies [6,47]. Its impact on mental health may be inversely associated with the inadequate access to necessary physical and mental health resources and/or unequal exposure to health risks that are often features of more disadvantaged neighborhoods. Many studies have described the importance of the neighborhood built environment on resident mental health [6] which is often integral to neighborhood affluence such that less affluent neighborhoods are also less likely to have access to public outdoor spaces, safe, well-maintained housing and transportation, or local health resources like clinics and hospitals [48].

African Americans in Tract and mental health show a positive linear relationship, suggesting that rates of poor mental health increases uniformly with increasing African Americans in tract. Hispanics or Latinos in Tract and mental health show a similar positive linear relationship, though weaker. African Americans in Tract and Hispanics or Latinos in Tract have been explored in a few past studies as ‘racial congruence’ or ‘ethnic diversity’, and so forth, though the conclusions vary [6]. For instance, some studies report that prevalence rates of depression are lower in neighborhoods with higher racial congruence [49] and community ethnic identification [50]. However, this relationship becomes inconsistent after accounting for individual-level socioeconomic factors [51]. As many of the subgroups that comprise this factor relate to affluence as well (e.g., unemployment and the amount of government assistance drawn), this factor is also, unsurprisingly, negatively correlated with Affluence. Minority groups are typically more likely to live in disadvantaged neighborhoods with less access to built-environment neighborhood features, which may be influenced by policies of disinvestment and structural barriers to the acquisition of wealth [52], and this disadvantage may contribute to the risk for mental health disparities [6]. Our study included factors describing characteristics of race and ethnic background because it is important to recognize the racial disparities prevalent in the United States. In describing these characteristics and their associations with mental health, the authors do not suggest that the disparity is caused by a difference in the physiology or genetics of different races. Rather, we aim to highlight health inequalities in communities of color described in past studies, such as a lack of resources in minority communities and the social discrimination against minority groups [53].

The relationship discovered between Singletons in Tract and mental health by our bivariate approach is weak, and our multivariate approach found no unique variance was explained by Singletons in Tract beyond the ACS variables age, sex, race, ethnicity, and poverty. This is despite previous research linking some of the characteristics of this factor to mental health. Singletons in Tract is representative, to an extent, of living arrangements, marital status, and residential mobility or neighborhood stability. Living alone has been reported to increase likelihood of depressive symptoms [54,55]. Marital status has also been shown to have an influence on mental health, married individuals being at lower risk of depression [56,57,58,59]. Additionally, greater residential mobility, including housing not owner-occupied and rates of moving in the last year derived from the ACS, has been associated with a decrease in mental health care visits for those with serious mental illness and comorbid medical conditions [6,60]. It is unclear why this relationship between these characteristics and mental health is not reflected at the neighborhood-level by our factor Singletons in Tract, however most of the studies we reference here describe individual-level effects which may not translate to the neighborhood level.

We discovered a very weak relationship between Seniors in Tract and mental health using our bivariate approach, however, our multivariate approach did pick up a medium effect size for this factor, suggesting Seniors in Tract may have some relationship to mental health rates. There are some studies that examine the relationship between neighborhood environment and rates of depression in an elderly cohort [6], however we have found no studies that examine the relationship between seniors in tract and rates of poor mental health. If this distinction is unclear, keep in mind that Seniors in Tract can also represent youth in tract, that is, a negative Seniors in Tract score implies that the tract is, on average, younger. When we compare Seniors in Tract to mental health, it is similar to comparing average neighborhood age to mental health. In the absence of neighborhood-level relationships, it may be enlightening to explore whether age is related to rates of poor mental health. The 2015 National Survey on Drug Use and Health (NSDUH) has shown that the prevalence of any mental illness among U.S. adults was highest in young adults aged 18–25 years, second highest in adults aged 26–49 years, and lowest in adults aged 50 years or older [61]. The gap in prevalence between these age groups has widened in the years 2015–2019 [61]. It is unclear why this relationship is not reflected at the neighborhood-level by our bivariate approach, however we may speculate that there is likely high variance in age within-tract, leading to a washing-out of individual-level effects.

### 4.3. Limitations

The variables used in this study were limited to those collected by the U.S. census. Consequently, there are some neighborhood characteristics shown in previous studies to be related to mental health that are not included in this study. For example, this study does not include walkability, neighborhood disorder, social factors, neighborhood hazards, the built environment, or the service environment [6]. Additionally, the mental health statistics from the BRFSS survey were only available for the largest 500 cities in the United States, and thus we were unable to examine the relationship between the factors and poor mental health rates in rural areas and small towns.

The 500 Cities SAE used data from the ACS to estimate health data. The measures included in the SAE were age, sex, race/ethnicity, and county-level poverty [33], leaving a possibility that confounding from age, race/ethnicity, and income measures may inflate the strength of the relationship between mental health and the neighborhood factors. We performed a multivariable analysis that demonstrated that when the variance explained by the ACS variables for age, sex, race, ethnicity, and poverty is removed from the model, all the factors apart from Singletons in Tract explain unique variance in the mental health statistic. We did not remove these ACS variables from our factor analysis because these variables are important to our discovered factor structure and improve the characterization of the neighborhood. Our intention is that our factor scores may be used in future studies, and they will be most useful when utilizing as many variables as possible. We also did not change our mental health variable because a nation-wide health survey sampled at the tract-level does not exist. The BRFSS is the largest continuously conducted multi-mode health survey system in the world [31] and still requires SAE to derive tract-level estimates, therefore SAE would be necessary to derive tract-level estimates for any health survey data in the U.S. It may be preferable to perform an SAE that excluded the ACS variables we use in our factor analysis, but we do not have the capability of performing our own SAE because we do not have access to the necessary individual-level data from the BRFSS. The mental health statistic we used in this study remains the best one for our purpose despite its limitations because it has a large sample population, has estimates available at the tract level, and demonstrates a clear relationship between poor mental health and our factors after removing the variance explained by confounding variables. Furthermore, the ACS data set serves as a reliable source for neighborhood statistics and remains the best data source for our factor analysis. The ACS statistics include responses from millions of households across the U.S., the data are collected consistently over time, and the statistics cover a broad range of characteristics.

There is also a potential for bias in the number of poor mental health days reported in the BRFSS, as this is a self-reported measure which could be influenced by cultural or social differences in how an individual defines a ‘poor mental health day’. While the mental health measure is a subjectively-defined variable, we have compared it to objectively-defined neighborhood factors. Perception of neighborhood conditions has been shown to be significantly correlated to rates of depression [6], but there is no assurance that this relationship does not simply depict the skewed outlook of those with depression. This is why, in the creation of the neighborhood factors, we avoid the use of neighborhood statistics derived from subjective resident response.

Additionally, there exist some challenges from an analysis standpoint. First, the reported data from the ACS has a margin of error that is not included in this analysis and may impact the factor structure. While this is a possibility, the similarity of the factor structure at the block group level demonstrates that the reduction of error, at least between the block group and tract levels, does not change the factor structure (see Comparison to Block Group, Supplementary Methods). Second, the indeterminacy problem is a well-known issue with factor analysis [62,63]. Factor analysis results in factors that must be subjectively defined. This has always been a fundamental problem of factor analysis. However, the circle plots clearly depict what each factor represents. Additionally, this problem is superseded by the utility of the latent variables over the raw data. The latent variable structure, though vague, simplifies interpretability drastically.

When considering the application of this tool, it is important to bear in mind that the factors reported here clearly describe neighborhood-level associations on the prevalence of mental health, but do not demonstrate these associations at the individual level. Thus, the influence of these factors at an individual level is still unknown. Neighborhood effects at the individual level are likely mediated by other influences, such as context of school or work. Jones et al. make a point that people experience substantial segregation across a range of spaces, such as areas of work or recreation, in their daily lives [64]. A study by Hermansen et al. found that after adjusting for family background, the effect of children’s neighborhood context on adult earnings later in life is near zero [65]. The extent to which the factor scores of an individual’s neighborhood affect their mental health must be explored in future studies.

There are some demographic differences between the BRFSS sample and the ACS sample, notably income, race, ethnicity, and age (see Table S39). Difference in the demographics of the two samples is possibly due to (1) more participants refusing to answer demographics questions in the BRFSS or (2) the exclusion of children younger than 18 in the BRFSS. Despite these demographic differences, both surveys are nation-wide samples taken by the Census and the CDC, and both surveys are some of the highest-quality representations of the United States population in the nation [31,66]. Furthermore, identical sample populations are not necessary to demonstrate a relationship between the neighborhood environment and characteristics of a particular cohort. We can use our factor scores to examine the relationship between neighborhood and any cohort of interest. We simply cannot say that the relationship exists outside the cohort studied. In our demonstration, the mental health characteristic is only measured for adults 18 years and older, and therefore we cannot make any conclusion on whether the neighborhood factors have a relationship to rates of poor mental health in children.

### 4.4. Future Directions

We have created a research tool intended to be implemented as an alternative to high-dimensional demographic data. We hope that this method for neighborhood characterization will facilitate research that will guide future clinical practice and public health initiatives. The code used to perform this analysis, as well as the factor scores for each tract, are available online for open use [36]. An interactive map of the factors is also available for visualization of the spatial distribution of each factor [67].

There are two clear levels of application at which the factors may be used. First, the factors may be applied to ascertain information on an individual’s environment. The factor scores of an individual’s neighborhood are easy to derive by using the Google Geocoding application programming interface (API) to convert an address to latitude longitude coordinates [68], then using the Area API from the U.S. Federal Communications Commission (FCC) to convert the coordinates to tracts [69]. This enables researchers to represent the neighborhood environment of their study population using five variables that are highly interpretable (intuitive loadings supported by prior research) and highly representative (60% of the variance accounted for by these factors). A paper by Feng et al. gives an example of how our factors may be used as independent variables [70].

Second, the factors may be applied at the community level, as demonstrated in this paper. At this level, the factors could guide larger scale policy, identifying communities to target resources. Though this study has shown no causal link between neighborhood factors and mental health, it has demonstrated a link nonetheless. This link indicates the need for mental health promotion in low income areas and minority communities.

## 5. Conclusions

The congruence of our findings and those demonstrated in prior research suggests that our factors are not only representative (accounting for 60% of the variance), but also interpretable as the loadings are intuitive and supported by the literature. The use of such factors in future research could offer a multidimensional approach to addressing similar questions regarding the impacts of neighborhoods on resident health. Furthermore, the relationship of our factors to interests of previous studies indicate that our factors are intuitively, as well as objectively, descriptive of neighborhoods.

Neighborhood factors based on census data provide comprehensive, objectively derived neighborhood characteristics, providing a useful tool for researchers, policymakers, and public health professionals to examine environmental factors impacting resident mental health. Information gained from these data could be used to monitor neighborhood trajectories and support changes in local policy or community-wide resources by providing insight regarding the areas in which intervention or a policy change would most strongly impact the residents of that area. Policymakers and public health professionals such as those in urban planning and social welfare can use this information to better understand how (1) the characteristics of their communities and (2) policies and programs (including those that may not appear directly related to health) have significant impacts on the health of its residents. To our knowledge, our work takes into consideration a variety of neighborhood statistics not previously explored, while remaining highly interpretable. We intend for these factors to be used to further explore the relationship between living environment and mental health. Our findings show that neighborhood characteristics are strongly related to mental health, indicating the importance of the factor model in future research focused on the influence of neighborhood characteristics on mental health.

## Figures and Tables

**Table 1:** Adjusting for Confounders of the Relationship Between Mental Health and the Neighborhood Factors.

Notation	Model	Variance Explained ${}^{1}$	Unique Variance Explained ${}^{2}$	Effect Size ${}^{3}$
$M_{a}$	ACS only	0.8376	-	-
$M_{a,1}$	ACS + Affluence	0.9037	0.0661	0.6861
$M_{a,2}$	ACS + Singletons in Tract	0.8376	0	0
$M_{a,3}$	ACS + Seniors in Tract	0.86	0.0224	0.1597
$M_{a,4}$	ACS + African Americans in Tract	0.8587	0.0211	0.149
$M_{a,5}$	ACS + Hispanics or Latinos in Tract	0.8768	0.0392	0.3177
$M_{a,1:5}$	ACS + All Neighborhood Factors	0.9177	0.0801	0.9728

${}^{1}$ The variance explained by the model is $R_{model}^{2}=\frac{V_{null}-V_{model}}{V_{null}}$. ${}^{2}$ The unique variance explained by the *i*th neighborhood factor was calculated as $R_{a,i}^{2}-R_{a}^{2}$. ${}^{3}$ The fixed effect size of the *i*th neighborhood factor is $f_{i}^{2}=\frac{R_{a,i}^{2}-R_{a}^{2}}{1-R_{a,i}^{2}}$.

## Data Availability

Publicly available datasets were analyzed in this study. This data can be found here: https://data.census.gov/, and here: https://chronicdata.cdc.gov/500-Cities-Places/500-Cities-Local-Data-for-Better-Health-2017-relea/vurf-k5wr.

## Supplementary Materials

The following are available online at https://www.mdpi.com/1660-4601/18/3/1202/s1, Figure S1: Block-Group Level 5-Factor Model Structure, Figure S2: Flow Chart Describing How Features Were Chosen, Figure S3: 4-Factor Model Structure, Figure S4: 6-Factor Model Structure, Figure S5: Scree Plot of Eigenvalues, Figure S6: The Affluence Factor Structure, Figure S7: The Singletons in Tract Factor Structure, Figure S8: The Seniors in Tract Factor Structure, Figure S9: The African Americans in Tract Factor Structure, Figure S10: The Hispanics or Latinos in Tract Factor Structure, Figure S11: Pearson Correlation Coefficients Between Factors, Figure S12: Boxplot of $R^{2}$ Values Across States by Factor, Table S1: B01001, Sex by age, Table S2: B02001, Race, Table S3: B03001, Hispanic or Latino by Specific Origin, Table S4: B05001, Nativity and Citizenship Status in the United States, Table S5: B07007, Geographical Mobility in the Past Year by Citizenship Status for Current Residence in the U.S., Table S6: B08301, Means of Transportation to Work, Table S7: B08302, Time Leaving Home to Go to Work, Table S8: B08303, Travel Time to Work, Table S9: B11001, Household Type (including Living Alone), Table S10: B12001, Sex by Marital Status for the Population 15 Years and over, Table S11: B15003, Educational Attainment for the Population 25 Years and Over, Table S12: B15012, Total Fields of Bachelor’s Degrees Reported, Table S13: B18101, Sex by Age by Disability Status, Table S14: B19001, Household Income in the Past 12 Months (in 2015 Inflation-Adjusted Dollars), Table S15: B19055, Social Security Income for Households, Table S16: B19056, Supplemental Security Income (SSI) for Households, Table S17: B19057, Public Assistance Income for Households, Table S18: B19058, Public Assistance Income or Food Stamps/SNAP in the Past 12 Months for Households, Table S19: B19059, Retirement Income for Households, Table S20: B19061, Aggregate Earnings in the Past 12 Months (in 2015 Inflation-Adjusted Dollars) for Households, Table S21: B19065, Aggregate Social Security Income in the Past 12 Months (in 2015 Inflation-Adjusted Dollars) for Households, Table S22. B19066, Aggregate Supplemental Security Income (Ssi) in the Past 12 Months (in 2015 Inflation-Adjusted Dollars) for Households, Table S23: B19067, Aggregate Public Assistance Income in the Past 12 Months (in 2015 Inflation-Adjusted Dollars) for Households, Table S24: B19069, Aggregate Retirement Income in the Past 12 Months (in 2015 Inflation-Adjusted Dollars) for Households, Table S25: B19301, Per Capita Income in the Past 12 Months (in 2015 Inflation-Adjusted Dollars), Table S26: B21001, SEX BY AGE BY VETERAN STATUS FOR THE CIVILIAN POPULATION 18 YEARS AND OVER, Table S27: B23025, Employment Status for the Population 16 Years and Over, Table S28: B25002, Occupancy Status, Table S29: B25003, Tenure, Table S30: B25018, Median Number of Rooms, Table S31: B25024, Units in Structure, Table S32: B25035, MEDIAN YEAR STRUCTURE BUILT, Table S33: B25040, House Heating Fuel, Table S34: B25051, KITCHEN FACILITIES FOR ALL HOUSING UNITS, Table S35: B25056, Contract Rent; Table S36: B25075, Value, Table S37: B25081, MORTGAGE STATUS, Table S38: Goodness-Of-Fit Statistics of Factor Analysis for 1 to 12 Factors, Table S39: Sample Demographic Characteristics.

## References

[1] Galster G., Hayes C., Johnson J. (2005). Identifying Robust, Parsimonious Neighborhood Indicators. J. Plan. Educ. Res..

[2] Arcaya M.C., Tucker-Seeley R.D., Kim R., Schnake-Mahl A., So M., Subramanian S. (2016). Research on neighborhood effects on health in the United States: A systematic review of study characteristics. Soc. Sci. Med..

[3] Meijer M., Röhl J., Bloomfield K., Grittner U. (2012). Do neighborhoods affect individual mortality? A systematic review and meta-analysis of multilevel studies. Soc. Sci. Med..

[4] Christian H., Zubrick S.R., Foster S., Giles-Corti B., Bull F., Wood L., Knuiman M., Brinkman S., Houghton S., Boruff B. (2015). The influence of the neighborhood physical environment on early child health and development: A review and call for research. Health Place.

[5] Truong K.D., Ma S. (2006). A systematic review of relations between neighborhoods and mental health. J. Ment. Health Policy Econ..

[6] Mair C.F., Diez Roux A.V., Galea S. (2008). Are Neighborhood Characteristics Associated with Depressive Symptoms? A Critical Review. J. Epidemiol. Community Health.

[7] Xue Y., Leventhal T., Brooks-Gunn J., Earls F.J. (2005). Neighborhood Residence and Mental Health Problems of 5- to 11-Year-Olds. Arch. Gen. Psychiatry.

[8] Cutrona C.E., Wallace G., Wesner K.A. (2006). Neighborhood Characteristics and Depression: An Examination of Stress Processes. Curr. Dir. Psychol. Sci..

[9] Eibner C., Sturn R., Gresenz C.R. (2004). Does relative deprivation predict the need for mental health services?. J. Ment. Health Policy Econ..

[10] American Psychological Association (2007). Task Force on Socioeconomic Status.

[11] Klijs B., Mendes de Leon C.F., Kibele E.U., Smidt N. (2017). Do social relations buffer the effect of neighborhood deprivation on health-related quality of life? Results from the LifeLines Cohort Study. Health Place.

[12] Moore K.A., Hirsch J.A., August C., Mair C., Sanchez B.N., Diez Roux A.V. (2016). Neighborhood Social Resources and Depressive Symptoms: Longitudinal Results from the Multi-Ethnic Study of Atherosclerosis. J. Urban Health.

[13] Van Dyck D., Teychenne M., McNaughton S.A., De Bourdeaudhuij I., Salmon J. (2015). Relationship of the Perceived Social and Physical Environment with Mental Health-Related Quality of Life in Middle-Aged and Older Adults: Mediating Effects of Physical Activity. PLoS ONE.

[14] Galea S., Ahern J., Rudenstine S., Wallace Z., Vlahov D. (2005). Urban built environment and depression: A multilevel analysis. J. Epidemiol. Community Health.

[15] Eibich P., Krekel C., Demuth I., Wagner G.G. (2016). Associations between Neighborhood Characteristics, Well-Being and Health Vary over the Life Course. Gerontology.

[16] Oakes J.M., Andrade K.E., Biyoow I.M., Cowan L.T. (2015). Twenty Years of Neighborhood Effect Research: An Assessment. Curr. Epidemiol. Rep..

[17] Campbell E., Henly J.R., Elliott D.S., Irwin K. (2009). Subjective Constructions of Neighborhood Boundaries: Lessons from a Qualitative Study of Four Neighborhoods. J. Urban Aff..

[18] Barnes J., Belsky J., Frost M., Melhuish E. (2011). Neighborhood characteristics and mental health: The relevance for mothers of infants in deprived English neighborhoods. Soc. Psychiatry Psychiatr. Epidemiol..

[19] Kolak M., Bhatt J., Park Y.H., Padrón N.A., Molefe A. (2020). Quantification of Neighborhood-Level Social Determinants of Health in the Continental United States. JAMA Netw. Open.

[20] Miles J.N., Weden M.M., Lavery D., Escarce J.J., Cagney K.A., Shih R.A. (2016). Constructing a Time-Invariant Measure of the Socio-economic Status of U.S. Census Tracts. J. Urban Health.

[21] Li Y.S., Chuang Y.C. (2009). Neighborhood Effects on an Individual’s Health Using Neighborhood Measurements Developed by Factor Analysis and Cluster Analysis. J. Urban Health.

[22] Mode N.A., Evans M.K., Zonderman A.B. (2016). Race, Neighborhood Economic Status, Income Inequality and Mortality. PLoS ONE.

[23] Centers for Disease Control and Prevention (2014). About BRFSS. https://www.cdc.gov/brfss/about/index.htm.

[24] Centers for Disease Control and Prevention (2017). 500 Cities: Local Data for Better Health. https://chronicdata.cdc.gov/500-Cities/500-Cities-Local-Data-for-Better-Health-2017-relea/vurf-k5wr.

[25] U.S. Census Bureau (2015). 2011–2015 American Community Survey 5-year Estimates.

[26] Torrieri N., ACSO, DSSD, SEHSD Program Staff (2014). American Community Survey Design and Methodology.

[27] Centers for Disease Control and Prevention (2018). BRFSS Frequently Asked Questions. https://www.cdc.gov/brfss/about/brfss_faq.htm.

[28] Centers for Disease Control and Prevention (2015). Behavioral Risk Factor Surveillance System. Overview: BRFSS 2014. https://www.cdc.gov/brfss/annual_data/2014/pdf/Overview_2014.pdf.

[29] Centers for Disease Control and Prevention (2015). Behavioral Risk Factor Surveillance System: 2014 Summary Data Quality Report. https://www.cdc.gov/brfss/annual_data/2014/pdf/2014_dqr.pdf.

[30] Centers for Disease Control and Prevention (2015). Behavioral Risk Factor Surveillance System: 2015 Summary Data Quality Report. https://www.cdc.gov/brfss/annual_data/2015/pdf/2015-sdqr.pdf.

[31] Centers for Disease Control and Prevention Behavioral Risk Factor Surveillance System History Fact Sheet. https://www.cdc.gov/brfss/factsheets/pdf/brfss-history.pdf.

[32] Zhang X., Holt J.B., Lu H., Wheaton A.G., Ford E.S., Greenlund K.J., Croft J.B. (2014). Multilevel Regression and Poststratification for Small-Area Estimation of Population Health Outcomes: A Case Study of Chronic Obstructive Pulmonary Disease Prevalence Using the Behavioral Risk Factor Surveillance System. Am. J. Epidemiol..

[33] Zhang X., Holt J.B., Yun S., Lu H., Greenlund K.J., Croft J.B. (2015). Validation of Multilevel Regression and Poststratification Methodology for Small Area Estimation of Health Indicators From the Behavioral Risk Factor Surveillance System. Am. J. Epidemiol..

[34] Selya A.S., Rose J.S., Dierker L.C., Hedeker D., Mermelstein R.J. (2012). A practical guide to calculating Cohen’s *f*^2^, a measure of local effect size, from PROC MIXED. Front. Psychol..

[35] R Core Team (2020). R: A Language and Environment for Statistical Computing. https://www.R-project.org.

[36] Forthman K.L. (2020). Neighborhood_analysis. https://github.com/kforthman/neighborhood_analysis.

[37] Glenn E.H. (2017). Acs: Download, Manipulate, and Present American Community Survey and Decennial Data from the US Census. https://CRAN.R-project.org/package=acs.

[38] Meyer D., Dimitriadou E., Hornik K., Weingessel A., Leisch F. (2017). e1071: Misc Functions of the Department of Statistics, Probability Theory Group (Formerly: E1071), TU Wien. https://CRAN.R-project.org/package=e1071.

[39] Wickham H., Seidel D. (2017). Scales: Scale Functions for Visualization. https://CRAN.R-project.org/package=scales.

[40] Torgo L. (2010). Data Mining with R, Learning with Case Studies. http://www.dcc.fc.up.pt/~ltorgo/DataMiningWithR.

[41] Revelle W. (2017). Psych: Procedures for Psychological, Psychometric, and Personality Research. https://CRAN.R-project.org/package=psych.

[42] Kelley-Moore J.A., Cagney K.A., Skarupski K.A., Everson-Rose S.A., Mendes de Leon C.F. (2016). Do Local Social Hierarchies Matter for Mental Health? A Study of Neighborhood Social Status and Depressive Symptoms in Older Adults. J. Gerontol. Ser. B.

[43] Mair C., Diez Roux A.V., Golden S.H., Rapp S., Seeman T., Shea S. (2015). Change in neighborhood environments and depressive symptoms in New York City: The Multi-Ethnic Study of Atherosclerosis. Health Place.

[44] Hu J., Kind A.J.H., Nerenz D. (2018). Area Deprivation Index Predicts Readmission Risk at an Urban Teaching Hospital. Am. J. Med Qual..

[45] Singh G.K. (2003). Area Deprivation and Widening Inequalities in US Mortality, 1969–1998. Am. J. Public Health.

[46] Borsboom D. (2006). When Does Measurement Invariance Matter?. Med. Care.

[47] Marmot M., Bell R. (2010). Health equity and development: The commission on social determinants of health. Eur. Rev..

[48] Wen M., Browning C.R., Cagney K.A. (2003). Poverty, affluence, and income inequality: Neighborhood economic structure and its implications for health. Soc. Sci. Med..

[49] Tweed D.L., Goldsmith H.F., Jackson D.J., Stiles D., Rae D.S., Kramer M. (1990). Racial congruity as a contextual correlate of mental disorder. Am. J. Orthopsychiatry.

[50] Simons R.L., Murry V., McLoyd V., Lin K.H., Cutrona C., Conger R.D. (2002). Discrimination, crime, ethnic identity, and parenting as correlates of depressive symptoms among African American children: A multilevel analysis. Dev. Psychopathol..

[51] Henderson C., Diez Roux A.V., Jacobs D.R., Kiefe C.I., West D., Williams D.R. (2005). Neighbourhood characteristics, individual level socioeconomic factors, and depressive symptoms in young adults: The CARDIA study. J. Epidemiol. Community Health.

[52] Solomon D., Maxwell C., Castro A. (2019). Systemic Inequality: Displacement, Exclusion, and Segregation.

[53] Bailey Z.D., Krieger N., Agénor M., Graves J., Linos N., Bassett M.T. (2017). Structural racism and health inequities in the USA: Evidence and interventions. Lancet.

[54] Stahl S.T., Beach S.R., Musa D., Schulz R. (2017). Living alone and depression: The modifying role of the perceived neighborhood environment. Aging Ment. Health.

[55] Xiu-Ying H., Qian C., Xiao-Dong P., Xue-Mei Z., Chang-Quan H. (2012). Living arrangements and risk for late life depression: A meta-analysis of published literature. Int. J. Psychiatry Med..

[56] Jang S.N., Kawachi I., Chang J., Boo K., Shin H.G., Lee H., Cho S.I. (2009). Marital status, gender, and depression: Analysis of the baseline survey of the Korean Longitudinal Study of Ageing (KLoSA). Soc. Sci. Med..

[57] Bulloch A.G., Williams J.V., Lavorato D.H., Patten S.B. (2009). The relationship between major depression and marital disruption is bidirectional. Depress. Anxiety.

[58] LaPierre T.A. (2009). Marital status and depressive symptoms over time: Age and gender variations. Fam. Relations.

[59] Yan X.Y., Huang S., Huang C.Q., Wu W.H., Qin Y. (2011). Marital status and risk for late life depression: A meta-analysis of the published literature. J. Int. Med. Res..

[60] Ku B.S., Lally C.A., Compton M.T., Druss B.G. (2020). Neighborhood Predictors of Outpatient Mental Health Visits Among Persons With Comorbid Medical and Serious Mental Illnesses. Psychiatr. Serv..

[61] U.S. Department of Health & Human Services (2020). 2019 NSDUH Annual National Report. https://www.samhsa.gov/data/sites/default/files/reports/rpt29393/2019NSDUHFFRPDFWHTML/2019NSDUHFFR1PDFW090120.pdf.

[62] Bollen K.A. (2002). Latent Variables in Psychology and the Social Sciences. Annu. Rev. Psychol..

[63] Steiger J.H. (1979). Factor indeterminacy in the 1930’s and the 1970’s some interesting parallels. Psychometrika.

[64] Jones M., Pebley A.R. (2014). Redefining Neighborhoods Using Common Destinations: Social Characteristics of Activity Spaces and Home Census Tracts Compared. Demography.

[65] Hermansen A.S., Borgen N.T., Mastekaasa A. (2020). Long-term trends in adult socio-economic resemblance between former schoolmates and neighbouring children. Eur. Sociol. Rev..

[66] U.S. Census Bureau American Community Survey: Coverage Rates. https://www.census.gov/acs/www/methodology/sample-size-and-data-quality/coverage-rates/.

[67] Forthman K.L. (2019). Factor_maps. https://kforthman.shinyapps.io/factor_maps.

[68] Google (2020). Geocoding API. https://developers.google.com/maps/documentation/geocoding/overview.

[69] U.S. Federal Communications Commission (2020). Area API. https://geo.fcc.gov/api/census.

[70] Feng C., Forthman K.L., Kuplicki R., Yeh H.w., Stewart J.L., Paulus M.P. (2019). Neighborhood affluence is not associated with positive and negative valence processing in adults with mood and anxiety disorders: A Bayesian inference approach. Neuroimage Clin..

