# Supplementary material for: Latent Variables Quantifying Neighborhood Characteristics and Their Associations with Poor Mental Health
Source: Int J Environ Res Public Health. 2021 Jan 29;18(3):1202. doi: 10.3390/ijerph18031202 (PMC7908478; doi:10.3390/ijerph18031202)
Supplement: Supplementary file 1 [file ijerph-18-01202-s001.pdf]

Supplementary Materials: Latent Variables Quantifying Neighborhood Characteristics and Their Associations with Poor Mental Health

Katherine L. Forthman [1,2], Janna M. Colaizzi [1], Hung-wen Yeh [1,3], Rayus Kuplicki [1] and Martin P. Paulus [1,*]

1. Supplementary Methods

1.1. Feature Selection

American Community Survey (ACS) data products include tables that contain estimates of demographic, social, economic, and housing characteristics. Tables are divided by type, subject, number, and iteration. An explanation of each division is given at [1]. A list of all available tables is available in the ACS documentation online [2]. The present study began by selecting all detailed type tables. Detailed tables provide basic estimates of demographics covered by the ACS. There are 1,045 detailed tables available for the 2011-2015 estimates. There are two subtypes within the detailed tables: collapsed and base tables. Base tables have a higher level of detail on estimates across topics. There are a total of 963 base tables available for 2011-2015 estimates. Tables are divided by subject, which describes the type of information included in the table. Several subjects were removed from consideration for this study, including: Sample (00), Ancestry (04), Place of Birth (06), Relationship to householder (09), Grandparents and Grandchildren Characteristics (10), Fertility (13), School enrollment (14), Language spoken at home (16), Poverty Status (17), Earnings (20), Food Stamps/Supplemental Nutrition Assistance Program (SNAP) (22), Industry, Occupation, and Class of Worker (24), Group Quarters (26), Health Insurance coverage (27), Computer and Internet use (28), Quality measures (98), and Allocation table for any Subject (99). These were removed either because they were considered redundant to another subject (06, 09, 14, 17, 20), or thought likely to overcomplicate the model without accounting for much of the variance (04, 10, 13, 16, 22, 24, 26 27). Subject 28 was not used because it is unavailable for the 2011-2015 estimates. Subjects 00, 98, and 99 were not used because they did not contain demographic data. Removing these subjects left us with 609 tables. Some tables were race iterated, i.e. stratified by racial categories. All such tables were removed, leaving 375 tables. Some tables are only available for Puerto Rico. These tables were also removed from consideration, leaving 347 tables. Within subject types, there is typically one table representing a particular demographic and the rest of the tables within the subject stratify this demographic (e.g. by sex, age, etc.). Wherever possible, we pulled the single unstratified table for each subject type. There were only 4 subject types for which we pulled more than one table: Commuting (Journey to Work); Place of Work (08), Educational Attainment; Undergraduate Field of Degree (15), Income (19), and Housing Characteristics (25). Multiple tables were pulled for these subjects because each represented a unique unstratified subset of the population. This left us with 37 tables. A list of the table IDs of all tables used in this study are given in Tables S1-37. Across all 37 tables, 461 statistics were included. A list of each statistic and how it was used in the analysis are given in Tables S1-37. Some statistics were removed for reasons given in Tables S1-37. A total of 246 statistics from the 37 tables were used in this study. These 246 statistics were collapsed into 39 features using the equations given in tables S1-37. This entire process is outlined in Figure S2.

1.2. Transformation

Most of the 39 statistics applied in factor analysis are proportions ranging between zero and one, inclusively. The nature of these measures causes left- and right-skewed distributions in raw statistics with modes on boundaries (zero or one). For example, there may be a small number of tracts where the characteristic highly represented, but in most tracts the feature is represented by only a small

fraction of the tract population. So that we can better distinguish the values, we desire to stretch the end of the distribution that is overrepresented without reordering the data. To do this, we developed a heuristic, data-driven transformation technique. A unique transformation was derived for each feature that minimized the absolute kurtosis of its distribution without changing the order of original values. The specific procedure is described as follows. Let x denote a raw tract statistic and t a strength parameter to be determined. Then x is transformed by a function

$$y(x;t) = \begin{cases} ln(x+t) & \text{if } x \text{ was skewed to right} \\ e^x - t & \text{if } x \text{ was skewed to left} \end{cases}$$

The transformation strength t was chosen via grid search independently for each feature to minimize the absolute value of the kurtosis for y. The grid was defined as:

$$\left\{ \frac{m * e^{0.1r} - 1}{e^5 - 1} \middle| r \, \epsilon \, 1, 2, 3, ..., 50 \right\}$$

where m was the maximum value of x observed. The transformed features were then scaled to have zero mean and unit variance. A feature was only transformed if the skewness factor was greater than 1 or less than -1. If the skewness factor was between -1 and 1, it was considered normal and thus did not need to be transformed.

1.3. Comparison to Block Group

Two definitions of the neighborhood were considered for this analysis. These were the two smallest census areas for which ACS data are provided: block groups and tracts. A block group consists of census blocks within a tract or block numbering area with the same first digit in their identification number. Block groups generally consist of 600–3,000 individuals and are smaller than tracts. Block groups are subdivisions of census tracts. While the block group characterizes a smaller area, it is important to note that it does not contain more data; that is, the statistics are derived from the same sample as the tract-level. The error margins for these smaller areas are larger. In addition, certain features of interest are unavailable at this scale. For certain questions that have a lower response rate, the error margins were too large, so the U.S. Census Bureau did not publish data at the block group level for these features. Such features included citizenship and mobility. These features were found to be important in the tract factor analysis. Specifically, rates of non-citizenship was one of the highest loading variables in the factor Hispanics or Latinos. The tract level factors were chosen in the interest of not sacrificing information. The tract has been used to define neighborhoods in a number of other comparable studies. Additionally, it was found that the factors derived for the block groups and tracts were nearly identical. This is shown in Figure S1.

2. Supplementary Figures

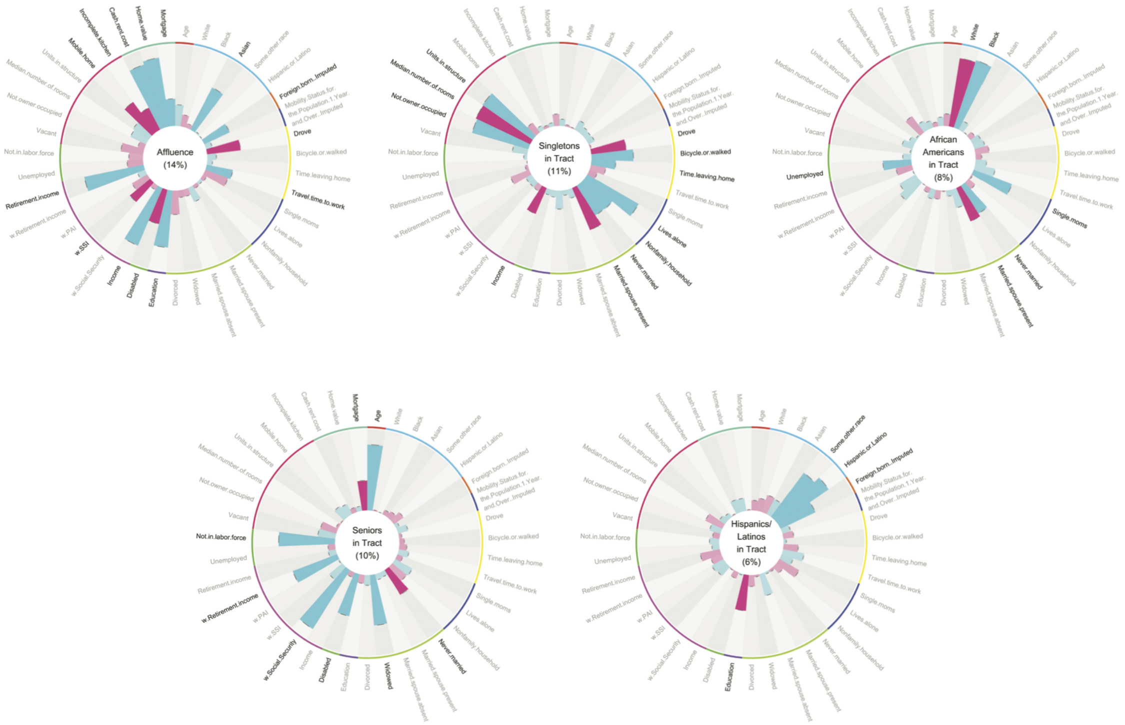

Figure S1. Block-Group Level 5-Factor Model Structure. The factors derived from block-group level statistics were very similar to the factors derived at the tract level.

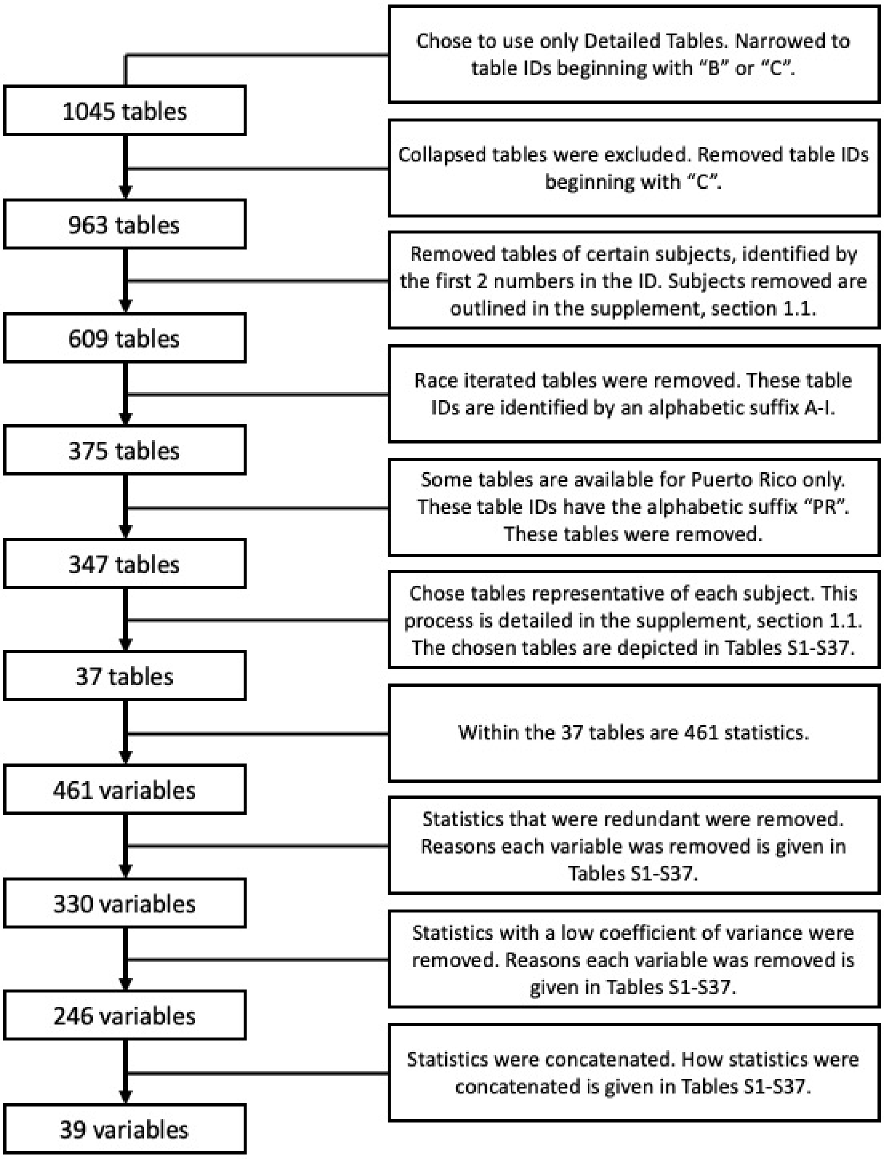

Figure S2. Flow Chart Describing How Features Were Chosen. Across all 37 tables, 461 statistics were included. A list of each statistic and how it was used in the analysis are given in Tables S1-37. Statistics were removed for reasons given in Tables S1-37. A total of 246 statistics from the 37 tables were used in this study. These 246 statistics were collapsed into 39 features using the equations given in tables S1-37.

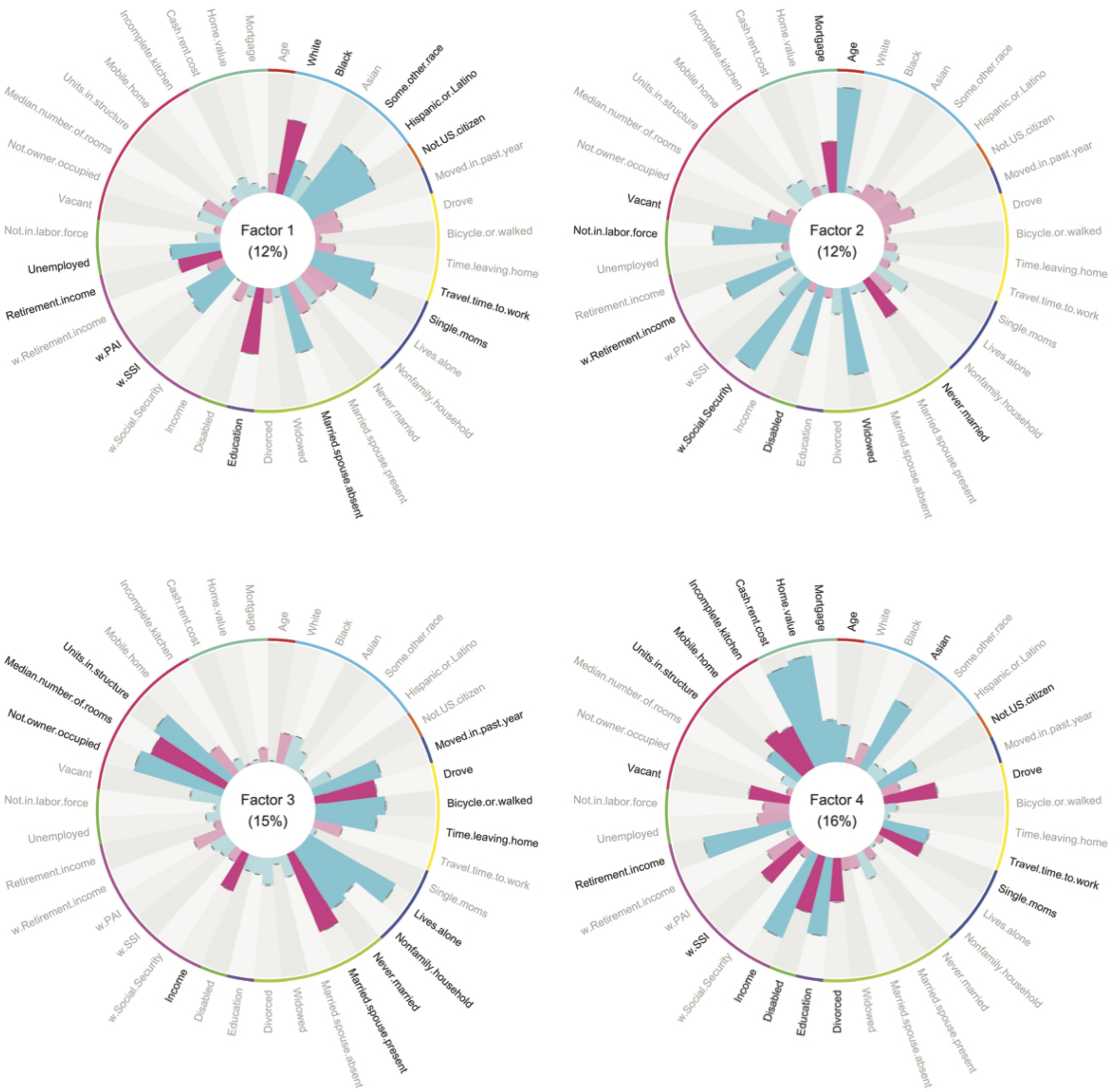

Figure S3. 4-Factor Model Structure. We explored a range between 1 and 12 factors and chose the factor number based on Kaiser's rule, a scree plot, the amount of total variance explained from each model produced, and the interpretability of the factor structure, shown here. The total variance explained by the 4-factor model is 0.554. This model is similar to the 5-factor model, but appears to combine the factors Hispanics or Latinos in Tract and African Americans in Tract in Factor 1.

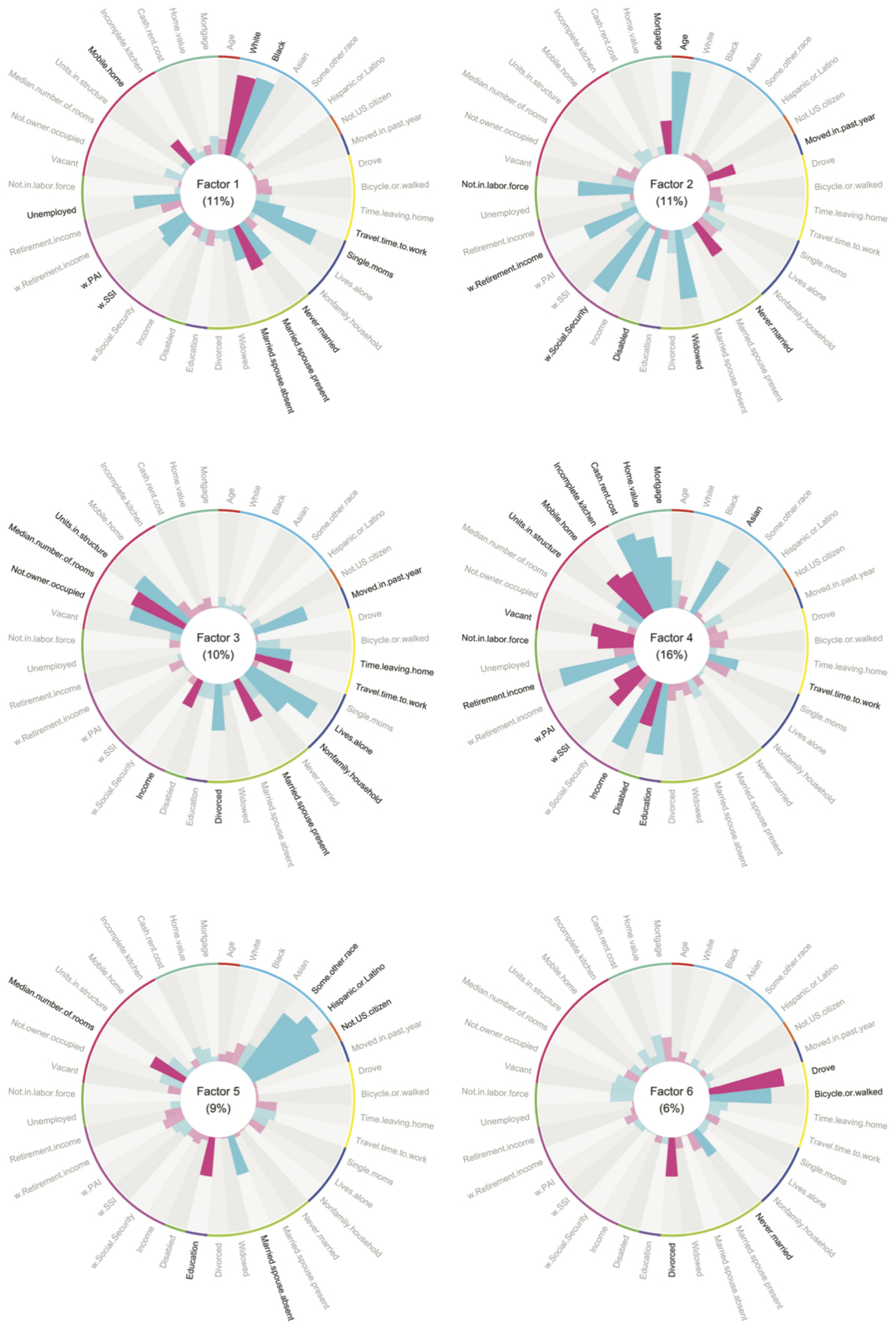

Figure S4. 6-Factor Model Structure. We explored a range between 1 and 12 factors and chose the factor number based on Kaiser's rule, a scree plot, the amount of total variance explained from each model produced, and the interpretability of the factor structure, shown here. The total variance explained by the 6-factor model is 0.633. The first five factors of this model are very similar to the 5-factor model. Factor 6 appears to describe tracts with higher rates of unmarried individuals and individuals who bike or walk to work, and it appears that Factor 3 and Factor 6 combined would describe the Singletons in Tract factor.

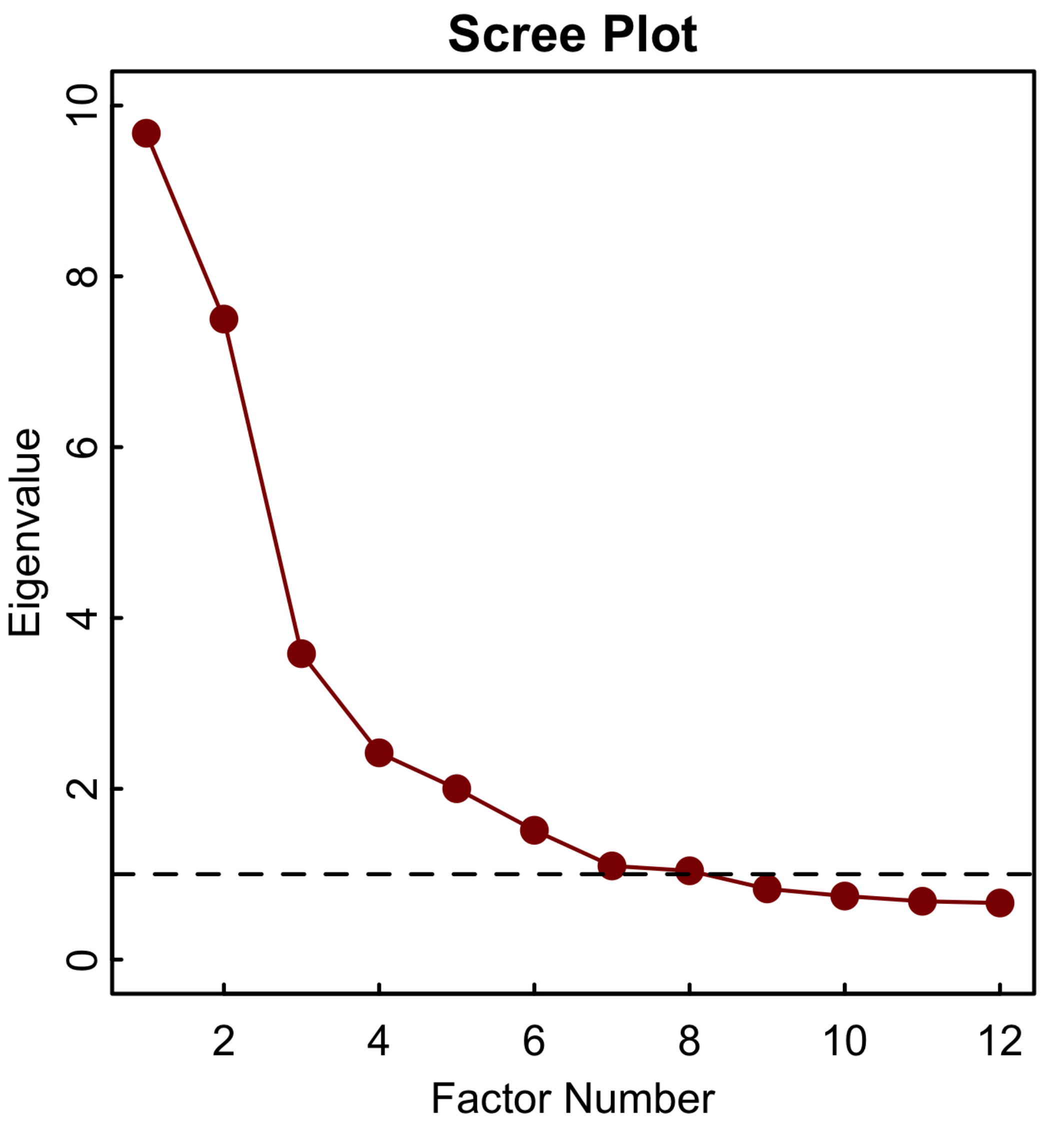

Figure S5. Scree Plot of Eigenvalues. This plot depicts the eigenvalue of each factor. A factor with an eigenvalue ≥ 1 describes more variance than any individual variable. Eight factors have an eigenvalue ≥ 1. The scree plot shows an 'elbow' at around five factors.

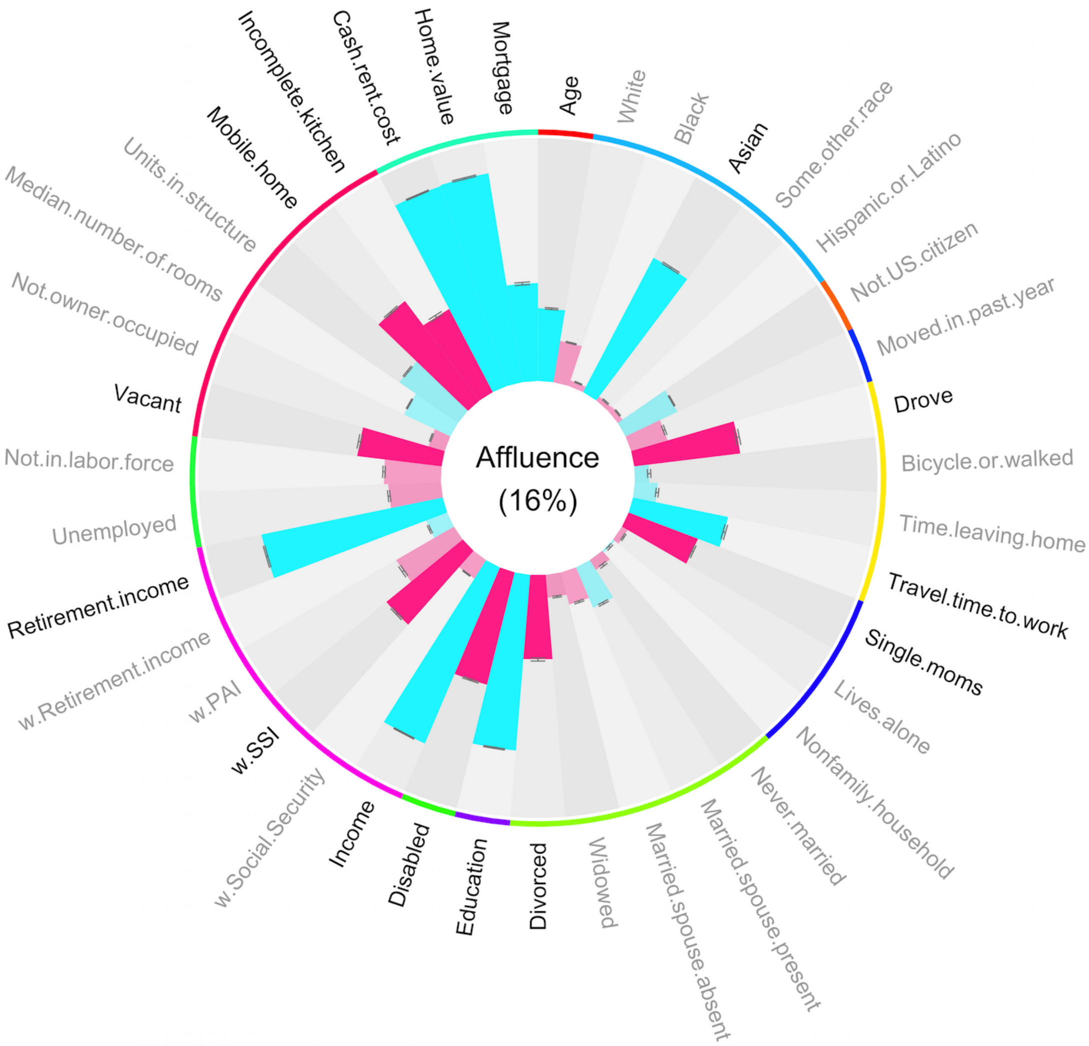

Figure S6. The Affluence Factor Structure. This is an enlarged version of the Affluence factor structure depicted in Figure 1. The highest loading variables in this factor are income (Income and Retirement.income), education (Education), and housing cost (Cash.rent.cost and Home.value). Also positively loading into this factor is Asian population (Asian), travel time to work (Travel.time.to.work), age (Age), and population with a mortgage (Mortgage). Negatively loading into this factor are population that drives to work (Drove), single moms (Single.moms), divorce rates (Divorced), disabled population (Disabled), rates of reliance on Supplemental Security Income (w.SSI), number of vacant homes (Vacant), population living in mobile homes (Mobile.home), and homes without complete kitchens (Incomplete.kitchen).

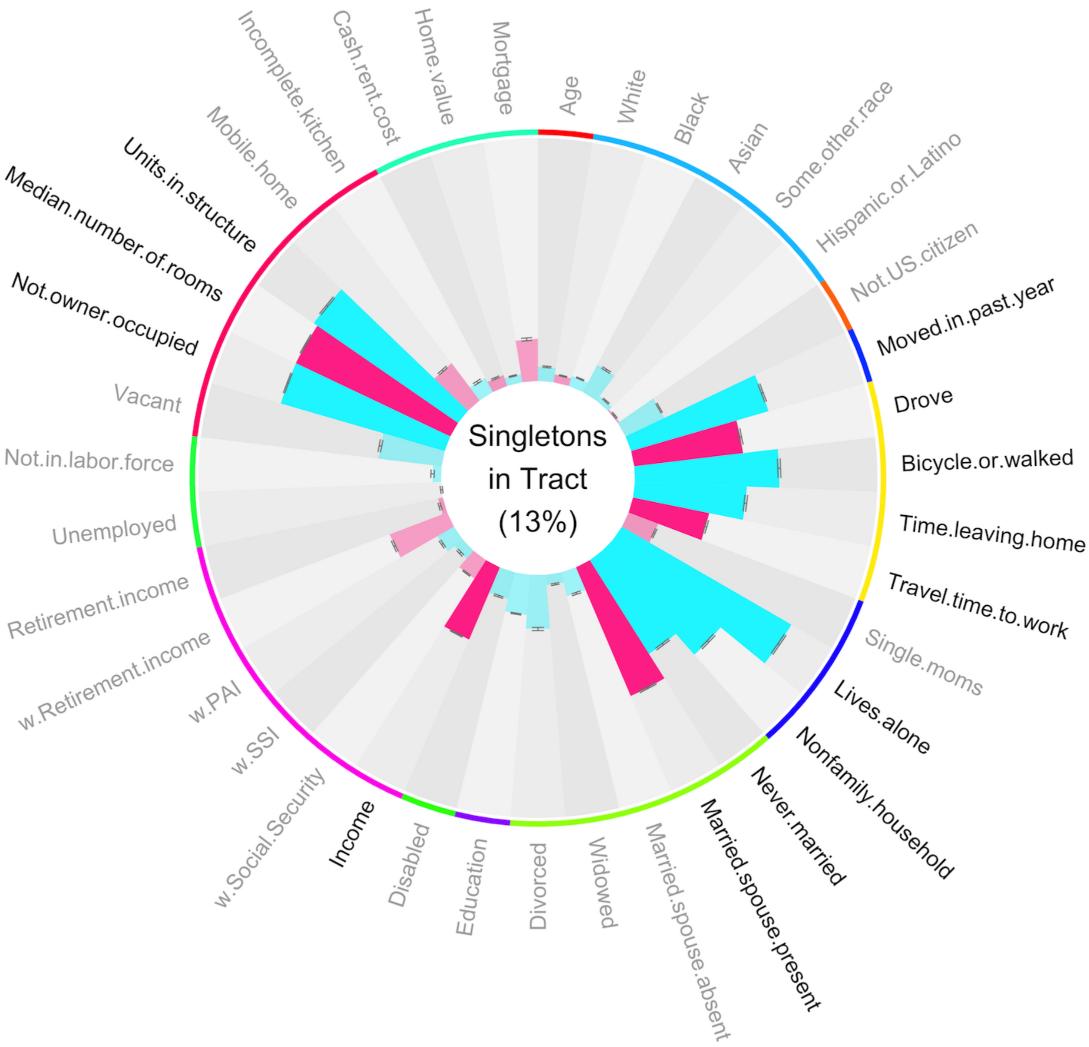

Figure S7. The Singletons in Tract Factor Structure. This is an enlarged version of the Singletons in Tract factor structure depicted in Figure 1. Single-person households and non-family households (Lives.alone and Nonfamily.household) load positively into this factor, implying that there are higher rates of individuals living alone or with roommates. Also loading strongly into this factor is living units per structure (Units.in.Structure), the median number of rooms per residence (Median.number.of.rooms), and residences not occupied by the owner (Not.owner.occupied), implying more apartment buildings in the tract. Also positively loading into this factor are population that has moved within the past year (Moved.in.past.year), the population that bikes or walks to work (Bicycle.or.walked), time leaving home for work (Time.leaving.home), and population that has never married (Never.married). Negatively loading into this factor are population that drives to work (Drove), travel time to work (Travel.time.to.work), population that is married and living with their spouse (Married.spouse.present), and income (Income). It is notable that age (Age) does not load into this factor.

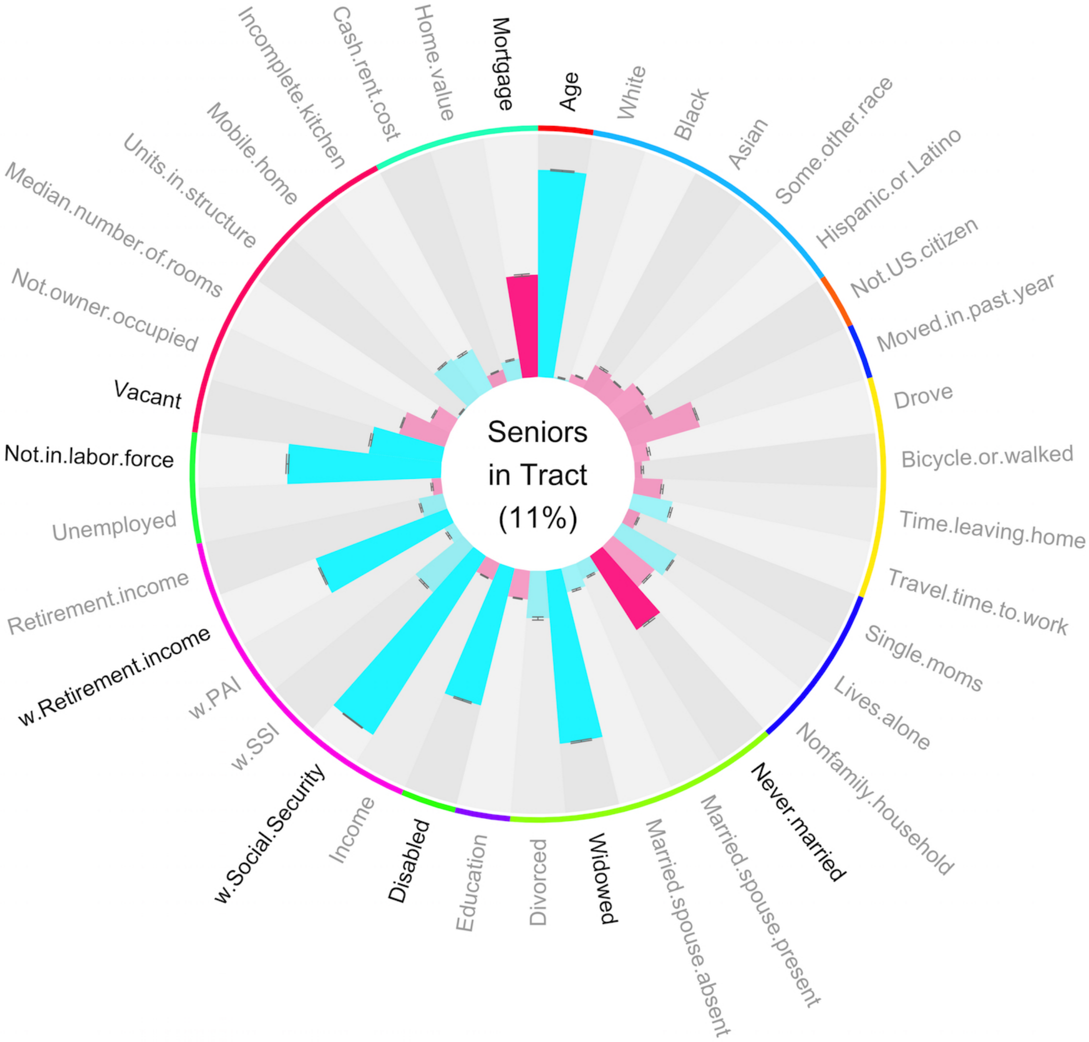

Figure S8. The Seniors in Tract Factor Structure. This is an enlarged version of the Seniors in Tract factor structure depicted in Figure 1. The highest loading variables in this factor are age (Age) and population receiving social security income (w.Social.Security), suggesting that tracts that score high on this factor have an older, retired population. Also loading positively into this factor are disabled and widowed populations (Disabled and Widowed), population out of the work force (Not.in.labor.force), population receiving retirement income (w.Retirement.income), and the number of vacant homes (Vacant). Negatively loading into this factor are population that has never been married (Never.married), and population with a mortgage (Mortgage).

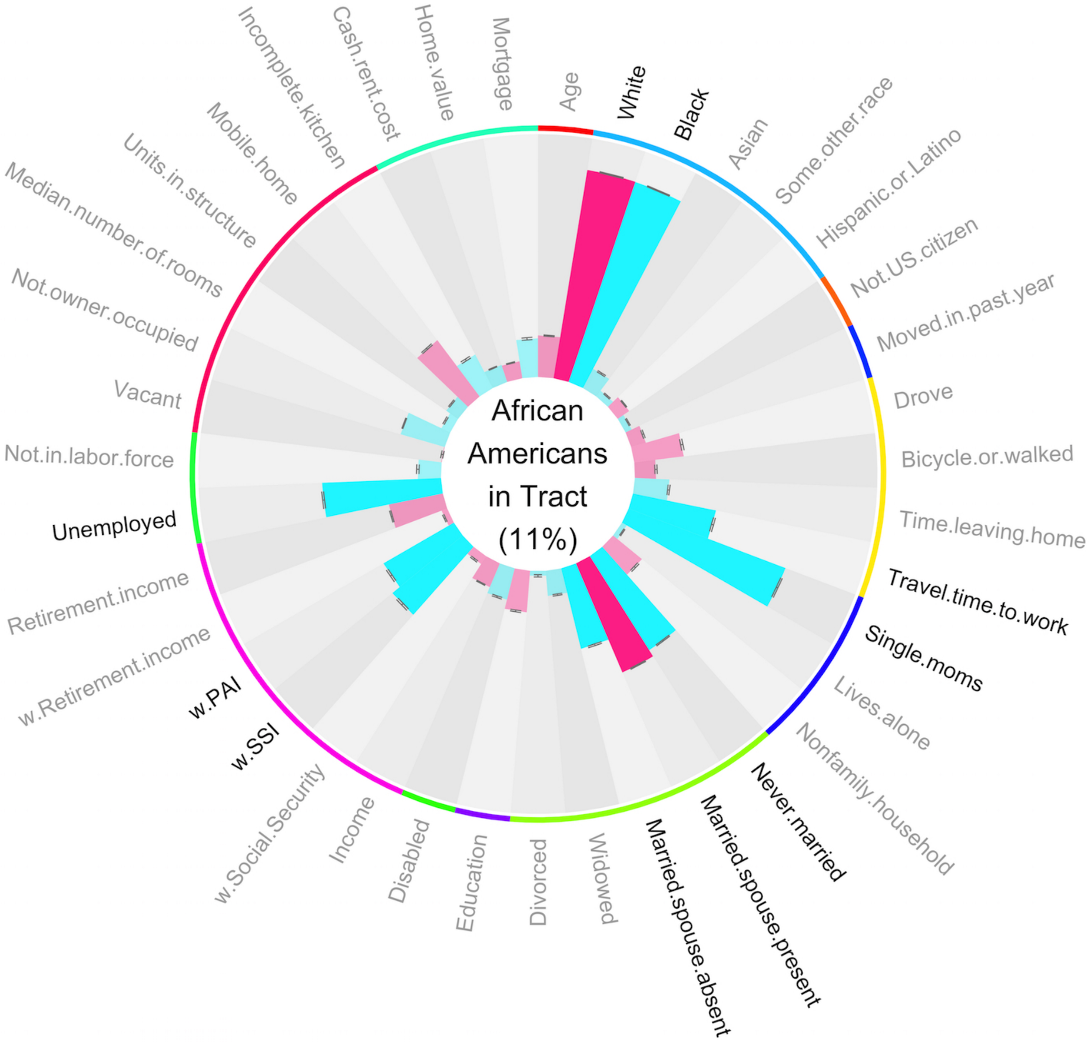

Figure S9. The African Americans in Tract Factor Structure. This is an enlarged version of the African Americans in Tract factor structure depicted in Figure 1. The highest loading variables in this factor are white population (White) and African American population (Black) suggesting that neighborhoods with a high African Americans in Tract have a high African American population and a low white population. These neighborhoods have higher rates of single mothers (Single.moms), unemployment (Unemployed), and reliance on Public Assistance Income (w.PAI) or Supplemental Security Income (w.SSI). Residents in these neighborhoods have longer commutes to work (Travel.time.to.work). There are also higher rates of unmarried individuals (Never.married) and individuals who are married but their spouse is absent (Married.spouse.absent).

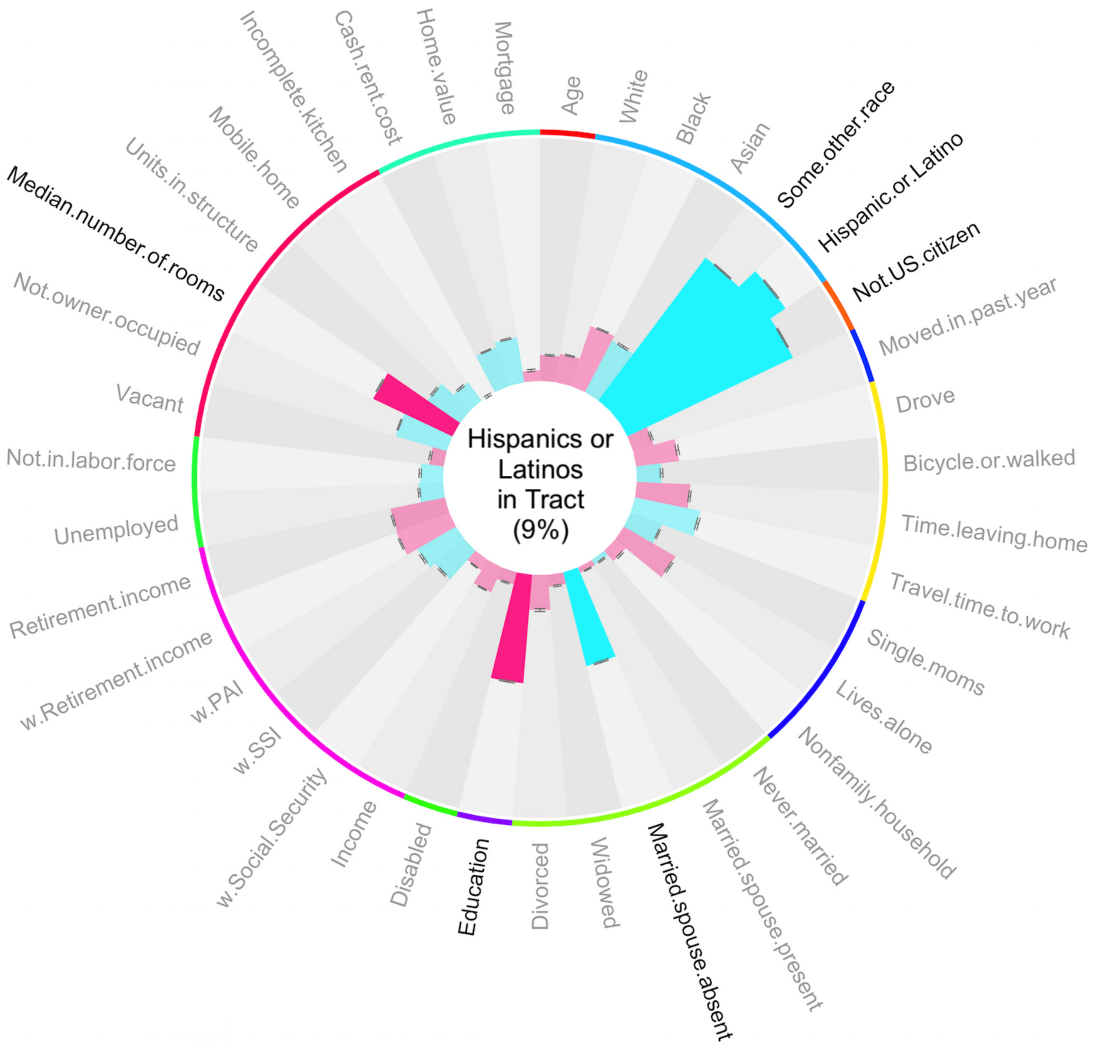

Figure S10. The Hispanics or Latinos in Tract Factor Structure. This is an enlarged version of the Hispanics or Latinos in Tract factor structure depicted in Figure 1. The highest loading variables in this factor are population with a race other than white, African American, or Asian (Some.other.race), population identifying as Hispanic or Latino (Hispanic.or.Latino), and population without U.S. citizenship (Not.US.citizen), suggesting that neighborhoods with a high Hispanics or Latinos in Tract have a higher Hispanic population and higher rates of non-citizenship. On average, these neighborhoods also tend to have smaller dwellings (Median.number.of.rooms), residents with lower education (Education), and higher rates of marital separation (Married.spouse.absent).

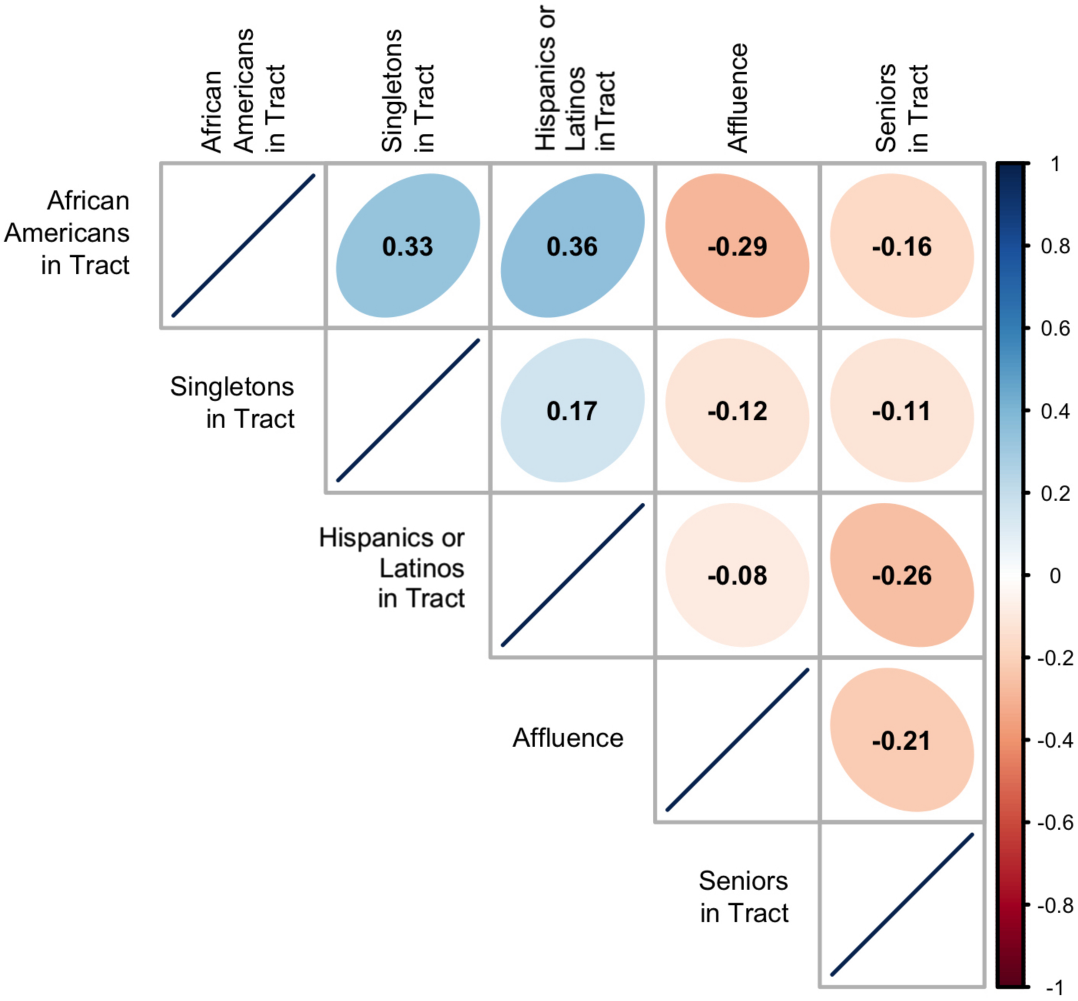

Figure S11. Pearson Correlation Coefficients Between Factors. Factor analysis does not assume that the latent variables are uncorrelated (unlike principle component analysis). Here is depicted the relationship between each of the factors in neighborhoods across the nation. It is notable that the two factors most highly associated with rates of poor mental health, Affluence and African Americans in Tract, are highly negatively correlated. The most correlated factors ($R > 0.3$) are Hispanics or Latinos in Tract and African Americans in Tract, and Singletons in Tract and African Americans in Tract.

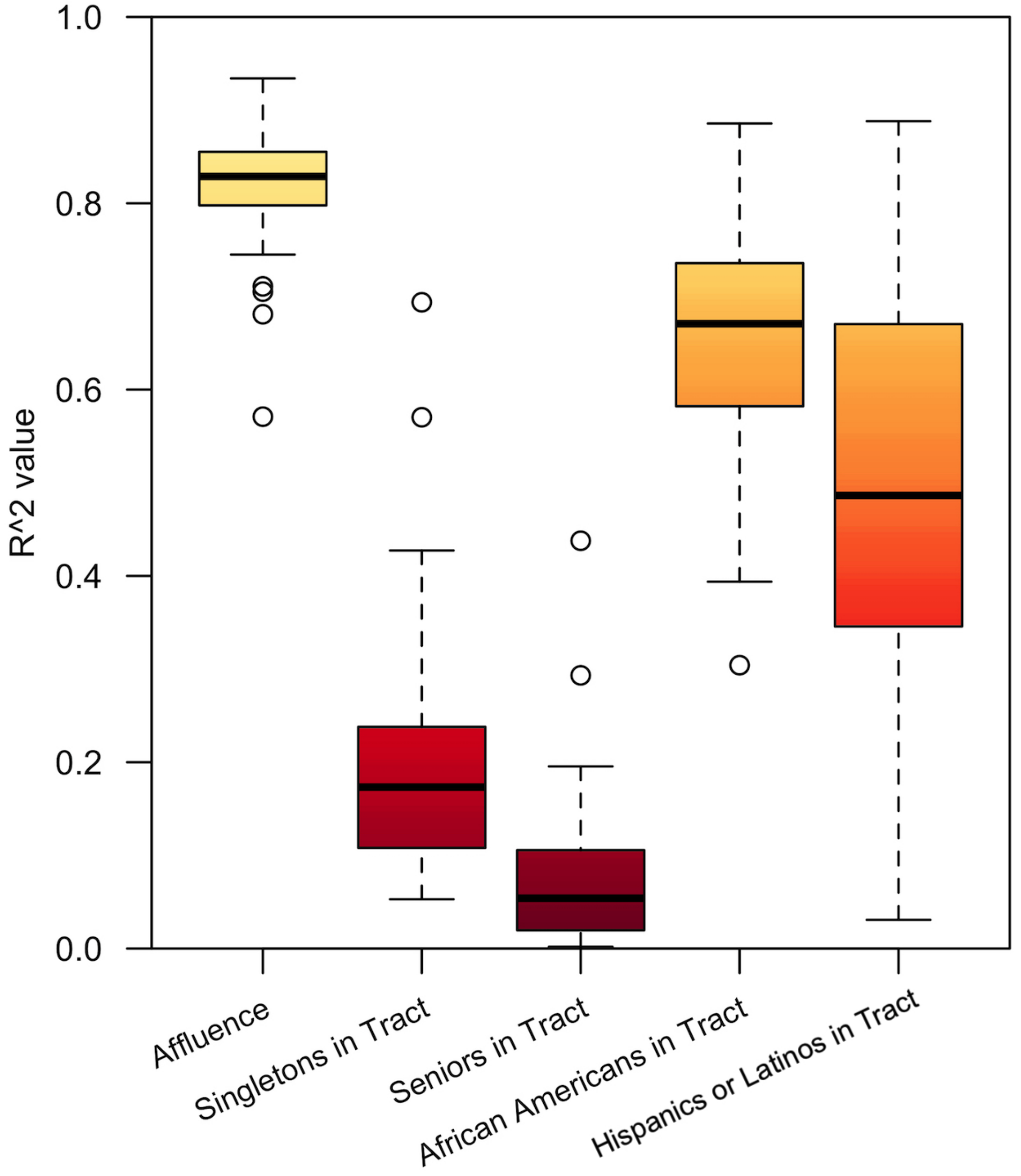

Figure S12. Boxplot of R^2 Values Across States by Factor. These boxplots are presented as an alternative to the visualization in Figure 2. The boxplot displays the range of values for each state of the fit of the spline modeling the relationship between each factor and rates of poor mental health. The R^2 values were on average highest for the Affluence factor, and second highest for African Americans in Tract. The R^2 values were on average lowest for the factor Seniors in Tract and second lowest for Singletons in Tract. The factor Hispanics or Latinos in Tract had the largest variance of fit across states. The color gradient corresponds to that in Figure 2.

Table S1. B01001, Sex by age

**Unique ID**	**Stub**	**Used in**	**Formula**	**Reason not Used**
B01001_001	Total:			
B01001_002	Male:	Not used		Low coefficient of variation
B01001_026	Female:	Not used		Low coefficient of variation

B01001_003<br>B01001_004	Male:!!Under 5 years<br>Male:!!5 to 9 years			
B01001_005	Male:!!10 to 14 years			
B01001_006	Male:!!15 to 17 years			
B01001_007	Male:!!18 and 19 years			
B01001_008	Male:!!20 years			
B01001_009	Male:!!21 years			
B01001_010	Male:!!22 to 24 years			
B01001_011	Male:!!25 to 29 years			
B01001_012	Male:!!30 to 34 years			
B01001_013	Male:!!35 to 39 years			
B01001_014	Male:!!40 to 44 years			
B01001_015	Male:!!45 to 49 years			
B01001_016	Male:!!50 to 54 years			
B01001_017	Male:!!55 to 59 years			
B01001_018	Male:!!60 and 61 years			
B01001_019	Male:!!62 to 64 years			
B01001_020	Male:!!65 and 66 years			
B01001_021	Male:!!67 to 69 years			
B01001_022	Male:!!70 to 74 years			
B01001_023	Male:!!75 to 79 years			
B01001_024	Male:!!80 to 84 years			
B01001_025	Male:!!85 years and over	Age	((B01001_003 + B01001_027)*2.5 + (B01001_004 + B01001_028)*7 + … + (B01001_024 + B01001_048)*82 + (B01001_025 + B01001_049)*85) / B01001_001	
B01001_027	Female:!!Under 5 years			
B01001_028	Female:!!5 to 9 years			
B01001_029	Female:!!10 to 14 years			
B01001_030	Female:!!15 to 17 years			
B01001_031	Female:!!18 and 19 years			
B01001_032	Female:!!20 years			
B01001_033	Female:!!21 years			
B01001_034	Female:!!22 to 24 years			

B01001_035	Female:!!25 to 29 years			
B01001_036	Female:!!30 to 34 years			
B01001_037	Female:!!35 to 39 years			
B01001_038	Female:!!40 to 44 years			
B01001_039	Female:!!45 to 49 years			
B01001_040	Female:!!50 to 54 years			
B01001_041	Female:!!55 to 59 years			
B01001_042	Female:!!60 and 61 years			
B01001_043	Female:!!62 to 64 years			
B01001_044	Female:!!65 and 66 years			
B01001_045	Female:!!67 to 69 years			
B01001_046	Female:!!70 to 74 years			
B01001_047	Female:!!75 to 79 years			
B01001_048	Female:!!80 to 84 years			
B01001_049	Female:!!85 years and over			

Table S2. B02001, Race

**Unique ID**	**Stub**	**Used in**	**Formula**	**Reason not Used**
B02001_001	Total:			
B02001_002	White alone	White	B02001_002 / B02001_001	
B02001_003	Black or African American alone	Black	B02001_003 / B02001_001	
B02001_005	Asian alone	Asian	B02001_005 / B02001_001	
B02001_004	American Indian and Alaska Native alone	Some.other.race	(B02001_004 + B02001_006 + B02001_007) / B02001_001	
B02001_006	Native Hawaiian and Other Pacific Islander alone			
B02001_007	Some other race alone			
B02001_008	Two or more races:	Not used		Low coefficient of variation
B02001_009	Two or more races:!!Two races including Some other race	Not used		Represented in another variable
B02001_010	Two or more races:!!Two races excluding Some other race, and three or more races	Not used		Represented in another variable

Table S3. B03001, Hispanic or Latino by Specific Origin

Unique ID	Stub	Used in	Formula	Reason not Used
B03001_001	Total:			
B03001_002	Not Hispanic or Latino	Not used		Represented in another variable
B03001_003	Hispanic or Latino:	Hispanic.or.Latino	B03001_003 / B03001_001	
B03001_004	Hispanic or Latino:!!Mexican	Not used		Represented in another variable
B03001_005	Hispanic or Latino:!!Puerto Rican	Not used		Represented in another variable
B03001_006	Hispanic or Latino:!!Cuban	Not used		Represented in another variable
B03001_007	Hispanic or Latino:!!Dominican (Dominican Republic)	Not used		Represented in another variable
B03001_008	Hispanic or Latino:!!Central American:	Not used		Represented in another variable
B03001_009	Hispanic or Latino:!!Central American:!!Costa Rican	Not used		Represented in another variable
B03001_010	Hispanic or Latino:!!Central American:!!Guatemalan	Not used		Represented in another variable
B03001_011	Hispanic or Latino:!!Central American:!!Honduran	Not used		Represented in another variable
B03001_012	Hispanic or Latino:!!Central American:!!Nicaraguan	Not used		Represented in another variable
B03001_013	Hispanic or Latino:!!Central American:!!Panamanian	Not used		Represented in another variable
B03001_014	Hispanic or Latino:!!Central American:!!Salvadoran	Not used		Represented in another variable
B03001_015	Hispanic or Latino:!!Central American:!!Other Central American	Not used		Represented in another variable

B03001_016	Hispanic or Latino:!!South American:	Not used		Represented in another variable
B03001_017	Hispanic or Latino:!!South American:!!Argentinean	Not used		Represented in another variable
B03001_018	Hispanic or Latino:!!South American:!!Bolivian	Not used		Represented in another variable
B03001_019	Hispanic or Latino:!!South American:!!Chilean	Not used		Represented in another variable
B03001_020	Hispanic or Latino:!!South American:!!Colombian	Not used		Represented in another variable
B03001_021	Hispanic or Latino:!!South American:!!Ecuadorian	Not used		Represented in another variable
B03001_022	Hispanic or Latino:!!South American:!!Paraguayan	Not used		Represented in another variable
B03001_023	Hispanic or Latino:!!South American:!!Peruvian	Not used		Represented in another variable
B03001_024	Hispanic or Latino:!!South American:!!Uruguayan	Not used		Represented in another variable
B03001_025	Hispanic or Latino:!!South American:!!Venezuelan	Not used		Represented in another variable
B03001_026	Hispanic or Latino:!!South American:!!Other South American	Not used		Represented in another variable
B03001_027	Hispanic or Latino:!!Other Hispanic or Latino:	Not used		Represented in another variable
B03001_028	Hispanic or Latino:!!Other Hispanic or Latino:!!Spaniard	Not used		Represented in another variable
B03001_029	Hispanic or Latino:!!Other Hispanic or Latino:!!Spanish	Not used		Represented in another variable

B03001_030	Hispanic or Latino:!!Other Hispanic or Latino:!!Spanish American	Not used		Represented in another variable
B03001_031	Hispanic or Latino:!!Other Hispanic or Latino:!!All other Hispanic or Latino	Not used		Represented in another variable

Table S4. B05001, Nativity and Citizenship Status in the United States

Unique ID	Stub	Used in	Formula	Reason not Used
B05001_001	Total:			
B05001_002	U.S. citizen, born in the United States	Not used		Represented in another variable
B05001_003	U.S. citizen, born in Puerto Rico or U.S. Island Areas	Not used		Represented in another variable
B05001_004	U.S. citizen, born abroad of American parent(s)	Not used		Represented in another variable
B05001_005	U.S. citizen by naturalization	Not used		Represented in another variable
B05001_006	Not a U.S. citizen	Not.US.citizen	B05001_006 / B05001_001	

Table S5. B07007, Geographical Mobility in the Past Year by Citizenship Status for Current Residence in the U.S.

Unique ID	Stub	Used in	Formula	Reason not Used
B07007_001	Total:			
B07007_002	Native	Not used		Represented in another variable
B07007_003	Foreign born:	Not used		Represented in another variable
B07007_004	Naturalized U.S. citizen	Not used		Represented in another variable
B07007_005	Not a U.S. citizen	Not used		Represented in another variable
B07007_006	Same house 1 year ago:	Moved.in.past.year	1 - (B07007_006 / B07007_001)	
B07007_007	Same house 1 year ago:!!Native	Not used		Represented in another variable
B07007_008	Same house 1 year ago:!!Foreign born:	Not used		Represented in another variable
B07007_009	Same house 1 year ago:!!Foreign born:!!Naturalized U.S. citizen	Not used		Represented in another variable
B07007_010	Same house 1 year ago:!!Foreign born:!!Not a U.S. citizen	Not used		Represented in another variable
B07007_011	Moved within same county:	Not used		Represented in another variable
B07007_012	Moved within same county:!!Native	Not used		Represented in another variable
B07007_013	Moved within same county:!!Foreign born:	Not used		Represented in another variable
B07007_014	Moved within same county:!!Foreign born:!!Naturalized U.S. citizen	Not used		Represented in another variable

B07007_015	Moved within same county:!!Foreign born:!!Not a U.S. citizen	Not used		Represented in another variable
B07007_016	Moved from different county within same state:	Not used		Represented in another variable
B07007_017	Moved from different county within same state:!!Native	Not used		Represented in another variable
B07007_018	Moved from different county within same state:!!Foreign born:	Not used		Represented in another variable
B07007_019	Moved from different county within same state:!!Foreign born:!!Naturalized U.S. citizen	Not used		Represented in another variable
B07007_020	Moved from different county within same state:!!Foreign born:!!Not a U.S. citizen	Not used		Represented in another variable
B07007_021	Moved from different state:	Not used		Represented in another variable
B07007_022	Moved from different state:!!Native	Not used		Represented in another variable
B07007_023	Moved from different state:!!Foreign born:	Not used		Represented in another variable
B07007_024	Moved from different state:!!Foreign born:!!Naturalized U.S. citizen	Not used		Represented in another variable
B07007_025	Moved from different state:!!Foreign born:!!Not a U.S. citizen	Not used		Represented in another variable
B07007_026	Moved from abroad:	Not used		Represented in another variable
B07007_027	Moved from abroad:!!Native	Not used		Represented in another variable

B07007_028	Moved from abroad:!!Foreign born:	Not used		Represented in another variable
B07007_029	Moved from abroad:!!Foreign born:!!Naturalized U.S. citizen	Not used		Represented in another variable
B07007_030	Moved from abroad:!!Foreign born:!!Not a U.S. citizen	Not used		Represented in another variable

Table S6. B08301, Means of Transportation to Work

Unique ID	Stub	Used in	Formula	Reason not Used
B08301_001	Total:			
B08301_002	Car, truck, or van:	Drove	(B08301_002 + B08301_017) / B08301_001	
B08301_017	Motorcycle			
B08301_003	Car, truck, or van:!!Drove alone	Not used		Represented in another variable
B08301_004	Car, truck, or van:!!Carpooled:	Not used		Represented in another variable
B08301_005	Car, truck, or van:!!Carpooled:!!In 2-person carpool	Not used		Represented in another variable
B08301_006	Car, truck, or van:!!Carpooled:!!In 3-person carpool	Not used		Represented in another variable
B08301_007	Car, truck, or van:!!Carpooled:!!In 4-person carpool	Not used		Represented in another variable
B08301_008	Car, truck, or van:!!Carpooled:!!In 5- or 6-person carpool	Not used		Represented in another variable
B08301_009	Car, truck, or van:!!Carpooled:!!In 7-or-more-person carpool	Not used		Represented in another variable
B08301_010	Public transportation (excluding taxicab):	Not used		Low coefficient of variation
B08301_011	Public transportation (excluding taxicab):!!Bus or trolley bus	Not used		Low coefficient of variation
B08301_012	Public transportation (excluding taxicab):!!Streetcar or trolley car (carro publico in Puerto Rico)	Not used		Low coefficient of variation
B08301_013	Public transportation (excluding taxicab):!!Subway or elevated	Not used		Low coefficient of variation
B08301_014	Public transportation (excluding taxicab):!!Railroad	Not used		Low coefficient of variation

B08301_015	Public transportation (excluding taxicab):!!Ferryboat	Not used		Low coefficient of variation
B08301_016	Taxicab	Not used		Low coefficient of variation
B08301_018	Bicycle	Bicycle.or.walked	(B08301_018 + B08301_019) / B08301_001	
B08301_019	Walked			
B08301_020	Other means	Not used		Low coefficient of variation
B08301_021	Worked at home	Not used		Low coefficient of variation

Table S7. B08302, Time Leaving Home to Go to Work

Unique ID	Stub	Used in	Formula	Reason not Used
B08302_001	Total:			
B08302_002	12:00 a.m. to 4:59 a.m.	Time.leaving.home	(B08302_002*0 + B08302_003*5 + ... + B08302_014*12 + B08302_015*16) / B08302_001	
B08302_003	5:00 a.m. to 5:29 a.m.			
B08302_004	5:30 a.m. to 5:59 a.m.			
B08302_005	6:00 a.m. to 6:29 a.m.			
B08302_006	6:30 a.m. to 6:59 a.m.			
B08302_007	7:00 a.m. to 7:29 a.m.			
B08302_008	7:30 a.m. to 7:59 a.m.			
B08302_009	8:00 a.m. to 8:29 a.m.			
B08302_010	8:30 a.m. to 8:59 a.m.			
B08302_011	9:00 a.m. to 9:59 a.m.			
B08302_012	10:00 a.m. to 10:59 a.m.			
B08302_013	11:00 a.m. to 11:59 a.m.			
B08302_014	12:00 p.m. to 3:59 p.m.			
B08302_015	4:00 p.m. to 11:59 p.m.			

Table S8. B08303, Travel Time to Work

Unique ID	Stub	Used in	Formula	Reason not Used
B08303_001	Total:			
B08303_002	Less than 5 minutes	Travel.time.to.work	(B08303_002*3 + B08303_003*7 + ... + B08303_012*74.5 + B08303_013*90) / B08303_001	
B08303_003	5 to 9 minutes			
B08303_004	10 to 14 minutes			
B08303_005	15 to 19 minutes			
B08303_006	20 to 24 minutes			
B08303_007	25 to 29 minutes			
B08303_008	30 to 34 minutes			
B08303_009	35 to 39 minutes			
B08303_010	40 to 44 minutes			
B08303_011	45 to 59 minutes			
B08303_012	60 to 89 minutes			
B08303_013	90 or more minutes			

Table S9. B11001, Household Type (including Living Alone)

Unique ID	Stub	Used in	Formula	Reason not Used
B11001_001	Total:			
B11001_002	Family households:	Not used		Represented in another variable
B11001_003	Family households:!!Married-couple family	Not used		Represented in another variable
B11001_004	Family households:!!Other family:	Not used		Represented in another variable
B11001_005	Family households:!!Other family:!!Male householder, no wife present	Not used		Represented in another variable
B11001_006	Family households:!!Other family:!!Female householder, no husband present	Single.moms	B11001_006 / B11001_001	
B11001_007	Nonfamily households:	Not used		Represented in another variable
B11001_008	Nonfamily households:!!Householder living alone	Lives.alone	B11001_008 / B11001_001	
B11001_009	Nonfamily households:!!Householder not living alone	Nonfamily.household	B11001_009 / B11001_001	

Table S10. B12001, Sex by Marital Status for the Population 15 Years and over

Unique ID	Stub	Used in	Formula	Reason not Used
B12001_001	Total:			
B12001_002	Male:	Not used		Represented in another variable
B12001_011	Female:	Not used		Represented in another variable
B12001_003	Male:!!Never married	Never.married	(B12001_003 + B12001_012) / B12001_001	
B12001_012	Female:!!Never married			
B12001_004	Male:!!Now married:	Not used		Represented in another variable
B12001_013	Female:!!Now married:	Not used		Represented in another variable
B12001_005	Male:!!Now married:!!Married, spouse present	Married.spouse.present	(B12001_005 + B12001_014) / B12001_001	
B12001_014	Female:!!Now married:!!Married, spouse present			
B12001_006	Male:!!Now married:!!Married, spouse absent:	Married.spouse.absent	(B12001_006 + B12001_015) / B12001_001	
B12001_015	Female:!!Now married:!!Married, spouse absent:			
B12001_007	Male:!!Now married:!!Married, spouse absent:!!Separated	Not used		Represented in another variable
B12001_016	Female:!!Now married:!!Married, spouse absent:!!Separated	Not used		Represented in another variable

B12001_008	Male:!!Now married:!!Married, spouse absent:!!Other	Not used		Represented in another variable
B12001_017	Female:!!Now married:!!Married, spouse absent:!!Other	Not used		Represented in another variable
B12001_009	Male:!!Widowed	Widowed	(B12001_009 + B12001_018) / B12001_001	
B12001_018	Female:!!Widowed			
B12001_010	Male:!!Divorced	Divorced	(B12001_010 + B12001_019) / B12001_001	
B12001_019	Female:!!Divorced			

Table S11. B15003, Educational Attainment for the Population 25 Years and Over

**Unique ID**	**Stub**	**Used in**	**Formula**	**Reason not Used**
B15003_001	Total:			
B15003_002	No schooling completed	Education	((B15003_002 + . . . + B15003_016)*0 + (B15003_017 + . . . + B15003_021)*1 + (B15003_022 + . . . + B15003_025)*2) / B15003_001	
B15003_003	Nursery school			
B15003_004	Kindergarten			
B15003_005	1st grade			
B15003_006	2nd grade			
B15003_007	3rd grade			
B15003_008	4th grade			
B15003_009	5th grade			
B15003_010	6th grade			
B15003_011	7th grade			
B15003_012	8th grade			
B15003_013	9th grade			
B15003_014	10th grade			
B15003_015	11th grade			
B15003_016	12th grade, no diploma			
B15003_017	Regular high school diploma			
B15003_018	GED or alternative credential			
B15003_019	Some college, less than 1 year			
B15003_020	Some college, 1 or more years, no degree			
B15003_021	Associate's degree			
B15003_022	Bachelor's degree			
B15003_023	Master's degree			
B15003_024	Professional school degree			
B15003_025	Doctorate degree			

Table S12. B15012, Total Fields of Bachelor's Degrees Reported

Unique ID	Stub	Used in	Formula	Reason not Used
B15012_001	Total:			
B15012_002	Science and Engineering!!Computers, Mathematics and Statistics	Not used		Low coefficient of variation
B15012_003	Science and Engineering!!Biological, Agricultural, and Environmental Sciences	Not used		Low coefficient of variation
B15012_004	Science and Engineering!!Physical and Related Sciences	Not used		Low coefficient of variation
B15012_005	Science and Engineering!!Psychology	Not used		Low coefficient of variation
B15012_006	Science and Engineering!!Social Sciences	Not used		Low coefficient of variation
B15012_007	Science and Engineering!!Engineering	Not used		Low coefficient of variation
B15012_008	Science and Engineering!!Multidisciplinary Studies	Not used		Low coefficient of variation
B15012_009	Science and Engineering Related Fields	Not used		Low coefficient of variation
B15012_010	Business	Not used		Low coefficient of variation
B15012_011	Education	Not used		Low coefficient of variation
B15012_012	Arts, Humanities, and Other!!Literature and Languages	Not used		Low coefficient of variation

B15012_013	Arts, Humanities, and Other!!Liberal Arts and History	Not used		Low coefficient of variation
B15012_014	Arts, Humanities, and Other!!Visual and Performing Arts	Not used		Low coefficient of variation
B15012_015	Arts, Humanities, and Other!!Communications	Not used		Low coefficient of variation
B15012_016	Arts, Humanities, and Other!!Other	Not used		Low coefficient of variation

Table S13. B18101, Sex by Age by Disability Status

Unique ID	Stub	Used in	Formula	Reason not Used
B18101_001	Total:			
B18101_002	Male:	Not used		Represented in another variable
B18101_021	Female:	Not used		Represented in another variable
B18101_003	Male:!!Under 5 years:	Not used		Represented in another variable
B18101_006	Male:!!5 to 17 years:	Not used		Represented in another variable
B18101_009	Male:!!18 to 34 years:	Not used		Represented in another variable
B18101_012	Male:!!35 to 64 years:	Not used		Represented in another variable
B18101_015	Male:!!65 to 74 years:	Not used		Represented in another variable
B18101_018	Male:!!75 years and over:	Not used		Represented in another variable
B18101_022	Female:!!Under 5 years:	Not used		Represented in another variable
B18101_025	Female:!!5 to 17 years:	Not used		Represented in another variable
B18101_028	Female:!!18 to 34 years:	Not used		Represented in another variable
B18101_031	Female:!!35 to 64 years:	Not used		Represented in another variable
B18101_034	Female:!!65 to 74 years:	Not used		Represented in another variable
B18101_037	Female:!!75 years and over:	Not used		Represented in another variable

B18101_005	Male:!!Under 5 years:!!No disability	Not used		Represented in another variable
B18101_008	Male:!!5 to 17 years:!!No disability	Not used		Represented in another variable
B18101_011	Male:!!18 to 34 years:!!No disability	Not used		Represented in another variable
B18101_014	Male:!!35 to 64 years:!!No disability	Not used		Represented in another variable
B18101_017	Male:!!65 to 74 years:!!No disability	Not used		Represented in another variable
B18101_020	Male:!!75 years and over:!!No disability	Not used		Represented in another variable
B18101_024	Female:!!Under 5 years:!!No disability	Not used		Represented in another variable
B18101_027	Female:!!5 to 17 years:!!No disability	Not used		Represented in another variable
B18101_030	Female:!!18 to 34 years:!!No disability	Not used		Represented in another variable
B18101_033	Female:!!35 to 64 years:!!No disability	Not used		Represented in another variable
B18101_036	Female:!!65 to 74 years:!!No disability	Not used		Represented in another variable
B18101_039	Female:!!75 years and over:!!No disability	Not used		Represented in another variable

B18101_004	Male:!!Under 5 years:!!With a disability	Disabled	((B18101_004 + B18101_007 + B18101_010 + … + B18101_019) + (B18101_023 + B18101_026 + B18101_029 + … + B18101_038)) / B18101_001	
B18101_007	Male:!!5 to 17 years:!!With a disability			
B18101_010	Male:!!18 to 34 years:!!With a disability			
B18101_013	Male:!!35 to 64 years:!!With a disability			
B18101_016	Male:!!65 to 74 years:!!With a disability			
B18101_019	Male:!!75 years and over:!!With a disability			
B18101_023	Female:!!Under 5 years:!!With a disability			
B18101_026	Female:!!5 to 17 years:!!With a disability			
B18101_029	Female:!!18 to 34 years:!!With a disability			
B18101_032	Female:!!35 to 64 years:!!With a disability			
B18101_035	Female:!!65 to 74 years:!!With a disability			
B18101_038	Female:!!75 years and over:!!With a disability			

Table S14. B19001, Household Income in the Past 12 Months (in 2015 Inflation-Adjusted Dollars)

Unique ID	Stub	Used in	Formula	Reason not Used
B19001_001	Total:			
B19001_002	Less than $10,000	Income	(B19001_002*4999.5 + B19001_003*12499.5 + … + B19001_016*174999.5 + B19001_017*200000) / B19001_001	
B19001_003	$10,000 to $14,999			
B19001_004	$15,000 to $19,999			
B19001_005	$20,000 to $24,999			
B19001_006	$25,000 to $29,999			
B19001_007	$30,000 to $34,999			
B19001_008	$35,000 to $39,999			
B19001_009	$40,000 to $44,999			
B19001_010	$45,000 to $49,999			
B19001_011	$50,000 to $59,999			
B19001_012	$60,000 to $74,999			
B19001_013	$75,000 to $99,999			
B19001_014	$100,000 to $124,999			
B19001_015	$125,000 to $149,999			
B19001_016	$150,000 to $199,999			
B19001_017	$200,000 or more			

Table S15. B19055, Social Security Income for Households

Unique ID	Stub	Used in	Formula	Reason not Used
B19055_001	Total:			
B19055_002	With Social Security income	w.Social.Security	B19055_002 / B19055_001	
B19055_003	No Social Security income	Not used		Represented in another variable

Table S16. B19056, Supplemental Security Income (SSI) for Households

Unique ID	Stub	Used in	Formula	Reason not Used
B19056_001	Total:			
B19056_002	With Supplemental Security Income (SSI)	w.SSI	B19056_002 / B19056_001	
B19056_003	No Supplemental Security Income (SSI)	Not used		Represented in another variable

Table S17. B19057, Public Assistance Income for Households

Unique ID	Stub	Used in	Formula	Reason not Used
B19057_001	Total:			
B19057_002	With public assistance income	w.PAI	B19057_002 / B19057_001	
B19057_003	No public assistance income	Not used		Represented in another variable

Table S18. B19058, Public Assistance Income or Food Stamps/SNAP in the Past 12 Months for Households

Unique ID	Stub	Used in	Formula	Reason not Used
B19058_001	Total:			
B19058_002	With cash public assistance or Food Stamps/SNAP	Not used		Low coefficient of variation
B19058_003	No cash public assistance or Food Stamps/SNAP	Not used		Low coefficient of variation

Table S19. B19059, Retirement Income for Households

Unique ID	Stub	Used in	Formula	Reason not Used
B19059_001	Total:			
B19059_002	With retirement income	w.Retirement.income	B19059_002 / B19059_001	
B19059_003	No retirement income	Not used		Represented in another variable

Table S20. B19061, Aggregate Earnings in the Past 12 Months (in 2015 Inflation-Adjusted Dollars) for Households

Unique ID	Stub	Used in	Formula	Reason not Used
B19061_001	Aggregate earnings in the past 12 months (in 2015 Inflation-adjusted dollars)	Not used		Represented in another variable

Table S21. B19065, Aggregate Social Security Income in the Past 12 Months (in 2015 Inflation-Adjusted Dollars) for Households

Unique ID	Stub	Used in	Formula	Reason not Used
B19065_001	Aggregate Social Security income in the past 12 months (in 2015 Inflation-adjusted dollars)	Not used		Represented in another variable

Table S22. B19066, Aggregate Supplemental Security Income (Ssi) in the Past 12 Months (in 2015 Inflation-Adjusted Dollars) for Households

Unique ID	Stub	Used in	Formula	Reason not Used
B19066_001	Aggregate Supplemental Security Income (SSI) in the past 12 months (in 2015 Inflation-adjusted dollars)	Not used		Represented in another variable

Table S23. B19067, Aggregate Public Assistance Income in the Past 12 Months (in 2015 Inflation-Adjusted Dollars) for Households

Unique ID	Stub	Used in	Formula	Reason not Used
B19067_001	Aggregate public assistance income in the past 12 months (in 2015 Inflation-adjusted dollars)	Not used		Represented in another variable

Table S24. B19069, Aggregate Retirement Income in the Past 12 Months (in 2015 Inflation-Adjusted Dollars) for Households

Unique ID	Stub	Used in	Formula	Reason not Used
B19069_001	Aggregate retirement income in the past 12 months (in 2015 Inflation-adjusted dollars)	Retirement.income	B19069_001	

Table S25. B19301, Per Capita Income in the Past 12 Months (in 2015 Inflation-Adjusted Dollars)

Unique ID	Stub	Used in	Formula	Reason not Used
B19301_001	Per capita income in the past 12 months (in 2015 Inflation-adjusted dollars)	Not used		Represented in another variable

Table S26. B21001, SEX BY AGE BY VETERAN STATUS FOR THE CIVILIAN POPULATION 18 YEARS AND OVER

Unique ID	Stub	Used in	Formula	Reason not Used
B21001_001	Total:	Not used		Low coefficient of variation
B21001_002	Veteran	Not used		Low coefficient of variation
B21001_003	Nonveteran	Not used		Low coefficient of variation
B21001_004	Male:	Not used		Low coefficient of variation
B21001_005	Veteran	Not used		Low coefficient of variation
B21001_006	Nonveteran	Not used		Low coefficient of variation
B21001_007	Male:!!18 to 34 years:	Not used		Low coefficient of variation
B21001_008	Male:!!18 to 34 years:!!Veteran	Not used		Low coefficient of variation
B21001_009	Male:!!18 to 34 years:!!Nonveteran	Not used		Low coefficient of variation
B21001_010	Male:!!35 to 54 years:	Not used		Low coefficient of variation
B21001_011	Male:!!35 to 54 years:!!Veteran	Not used		Low coefficient of variation
B21001_012	Male:!!35 to 54 years:!!Nonveteran	Not used		Low coefficient of variation
B21001_013	Male:!!55 to 64 years:	Not used		Low coefficient of variation
B21001_014	Male:!!55 to 64 years:!!Veteran	Not used		Low coefficient of variation

B21001_015	Male:!!55 to 64 years:!!Nonveteran	Not used		Low coefficient of variation
B21001_016	Male:!!65 to 74 years:	Not used		Low coefficient of variation
B21001_017	Male:!!65 to 74 years:!!Veteran	Not used		Low coefficient of variation
B21001_018	Male:!!65 to 74 years:!!Nonveteran	Not used		Low coefficient of variation
B21001_019	Male:!!75 years and over:	Not used		Low coefficient of variation
B21001_020	Male:!!75 years and over:!!Veteran	Not used		Low coefficient of variation
B21001_021	Male:!!75 years and over:!!Nonveteran	Not used		Low coefficient of variation
B21001_022	Female:	Not used		Low coefficient of variation
B21001_023	Veteran	Not used		Low coefficient of variation
B21001_024	Nonveteran	Not used		Low coefficient of variation
B21001_025	Female:!!18 to 34 years:	Not used		Low coefficient of variation
B21001_026	Female:!!18 to 34 years:!!Veteran	Not used		Low coefficient of variation
B21001_027	Female:!!18 to 34 years:!!Nonveteran	Not used		Low coefficient of variation
B21001_028	Female:!!35 to 54 years:	Not used		Low coefficient of variation
B21001_029	Female:!!35 to 54 years:!!Veteran	Not used		Low coefficient of variation

B21001_030	Female:!!35 to 54 years:!!Nonveteran	Not used		Low coefficient of variation
B21001_031	Female:!!55 to 64 years:	Not used		Low coefficient of variation
B21001_032	Female:!!55 to 64 years:!!Veteran	Not used		Low coefficient of variation
B21001_033	Female:!!55 to 64 years:!!Nonveteran	Not used		Low coefficient of variation
B21001_034	Female:!!65 to 74 years:	Not used		Low coefficient of variation
B21001_035	Female:!!65 to 74 years:!!Veteran	Not used		Low coefficient of variation
B21001_036	Female:!!65 to 74 years:!!Nonveteran	Not used		Low coefficient of variation
B21001_037	Female:!!75 years and over:	Not used		Low coefficient of variation
B21001_038	Female:!!75 years and over:!!Veteran	Not used		Low coefficient of variation
B21001_039	Female:!!75 years and over:!!Nonveteran	Not used		Low coefficient of variation

Table S27. B23025, Employment Status for the Population 16 Years and Over

Unique ID	Stub	Used in	Formula	Reason not Used
B23025_001	Total:			
B23025_002	In labor force:	Not used		Represented in another variable
B23025_003	In labor force:!!Civilian labor force:	Not used		Represented in another variable
B23025_004	In labor force:!!Civilian labor force:!!Employed	Not used		Represented in another variable
B23025_005	In labor force:!!Civilian labor force:!!Unemployed	Unemployed	B23025_005 / B23025_001	
B23025_006	In labor force:!!Armed Forces	Not used		Represented in another variable
B23025_007	Not in labor force	Not.in.labor.force	B23025_007 / B23025_001	

Table S28. B25002, Occupancy Status

Unique ID	Stub	Used in	Formula	Reason not Used
B25002_001	Total:			
B25002_002	Occupied	Not used		Represented in another variable
B25002_003	Vacant	Vacant	B25002_003 / B25002_001	

Table S29. B25003, Tenure

Unique ID	Stub	Used in	Formula	Reason not Used
B25003_001	Total:			
B25003_002	Owner occupied	Not.owner.occupied	1 - (B25003_002 / B25003_001)	
B25003_003	Renter occupied	Not used		Represented in another variable

Table S30. B25018, Median Number of Rooms

Unique ID	Stub	Used in	Formula	Reason not Used
B25018_001	Median number of rooms	Median.number.of.rooms	B25018_001	

Table S31. B25024, Units in Structure

Unique ID	Stub	Used in	Formula	Reason not Used
B25024_001	Total:			
B25024_002	1, detached	Units.in.structure	(B25024_002*1 + B25024_003*1 + B25024_004*2 + . . . + B25024_008*34.5 + B25024_009*50) / B25024_001	
B25024_003	1, attached			
B25024_004	2			
B25024_005	3 or 4			
B25024_006	5 to 9			
B25024_007	10 to 19			
B25024_008	20 to 49			
B25024_009	50 or more			
B25024_010	Mobile home	Mobile.home	(B25024_010 + B25024_011) / B25024_001	
B25024_011	Boat, RV, van, etc.			

Table S32. B25035, MEDIAN YEAR STRUCTURE BUILT

Unique ID	Stub	Used in	Formula	Reason not Used
B25035_001	Median year structure built	Not used		Low coefficient of variation

Table S33. B25040, House Heating Fuel

Unique ID	Stub	Used in	Formula	Reason not Used
B25040_001	Total:			
B25040_002	Utility gas	Not used		Low coefficient of variation
B25040_003	Bottled, tank, or LP gas	Not used		Low coefficient of variation
B25040_004	Electricity	Not used		Low coefficient of variation
B25040_005	Fuel oil, kerosene, etc.	Not used		Low coefficient of variation
B25040_006	Coal or coke	Not used		Low coefficient of variation
B25040_007	Wood	Not used		Low coefficient of variation
B25040_008	Solar energy	Not used		Low coefficient of variation
B25040_009	Other fuel	Not used		Low coefficient of variation
B25040_010	No fuel used	Not used		Low coefficient of variation

Table S34. B25051, KITCHEN FACILITIES FOR ALL HOUSING UNITS

Unique ID	Stub	Used in	Formula	Reason not Used
B25051_001	Total:			
B25051_002	Complete kitchen facilities	Not used		Low coefficient of variation
B25051_003	Lacking complete kitchen facilities	Not used		Low coefficient of variation

Table S35. B25056, Contract Rent

Unique ID	Stub	Used in	Formula	Reason not Used
B25056_001	Total:			
B25056_002	With cash rent:	Cash.rent.cost	(B25056_003*49.5 + B25056_004*124.5 . . . + B25056_025*3249.5 + B25056_026*3500) / B25056_002	
B25056_003	With cash rent:!!Less than $100			
B25056_004	With cash rent:!!$100 to $149			
B25056_005	With cash rent:!!$150 to $199			
B25056_006	With cash rent:!!$200 to $249			
B25056_007	With cash rent:!!$250 to $299			
B25056_008	With cash rent:!!$300 to $349			
B25056_009	With cash rent:!!$350 to $399			
B25056_010	With cash rent:!!$400 to $449			
B25056_011	With cash rent:!!$450 to $499			
B25056_012	With cash rent:!!$500 to $549			
B25056_013	With cash rent:!!$550 to $599			
B25056_014	With cash rent:!!$600 to $649			
B25056_015	With cash rent:!!$650 to $699			
B25056_016	With cash rent:!!$700 to $749			
B25056_017	With cash rent:!!$750 to $799			
B25056_018	With cash rent:!!$800 to $899			
B25056_019	With cash rent:!!$900 to $999			
B25056_020	With cash rent:!!$1,000 to $1,249			
B25056_021	With cash rent:!!$1,250 to $1,499			
B25056_022	With cash rent:!!$1,500 to $1,999			
B25056_023	With cash rent:!!$2,000 to $2,499			
B25056_024	With cash rent:!!$2,500 to $2,999			
B25056_025	With cash rent:!!$3,000 to $3,499			
B25056_026	With cash rent:!!$3,500 or more			
B25056_027	No cash rent	Not used		Represented in another variable

Table S36. B25075, Value

Unique ID	Stub	Used in	Formula	Reason not Used
B25075_001	Total:			
B25075_002	Less than $10,000	Home.value	(B25075_002*4999.5 + B25075_003*12499.5 + … + B25075_026*1750000 + B25075_027*2000000) / B25075_001	
B25075_003	$10,000 to $14,999			
B25075_004	$15,000 to $19,999			
B25075_005	$20,000 to $24,999			
B25075_006	$25,000 to $29,999			
B25075_007	$30,000 to $34,999			
B25075_008	$35,000 to $39,999			
B25075_009	$40,000 to $49,999			
B25075_010	$50,000 to $59,999			
B25075_011	$60,000 to $69,999			
B25075_012	$70,000 to $79,999			
B25075_013	$80,000 to $89,999			
B25075_014	$90,000 to $99,999			
B25075_015	$100,000 to $124,999			
B25075_016	$125,000 to $149,999			
B25075_017	$150,000 to $174,999			
B25075_018	$175,000 to $199,999			
B25075_019	$200,000 to $249,999			
B25075_020	$250,000 to $299,999			
B25075_021	$300,000 to $399,999			
B25075_022	$400,000 to $499,999			
B25075_023	$500,000 to $749,999			
B25075_024	$750,000 to $999,999			
B25075_025	$1,000,000 to $1,499,999			
B25075_026	$1,500,000 to $1,999,999			
B25075_027	$2,000,000 or more			

Table S37. B25081, MORTGAGE STATUS

Unique ID	Stub	Used in	Formula	Reason not Used
B25081_001	Total:			
B25081_002	Housing units with a mortgage, contract to purchase, or similar debt:	Mortgage	B25081_002 / B25081_001	
B25081_003	Housing units with a mortgage, contract to purchase, or similar debt:!!With either a second mortgage or home equity loan, but not both:	Not used		Represented in another variable
B25081_004	Housing units with a mortgage, contract to purchase, or similar debt:!!With either a second mortgage or home equity loan, but not both:!!Second mortgage only	Not used		Represented in another variable
B25081_005	Housing units with a mortgage, contract to purchase, or similar debt:!!With either a second mortgage or home equity loan, but not both:!!Home equity loan only	Not used		Represented in another variable
B25081_006	Housing units with a mortgage, contract to purchase, or similar debt:!!Both second mortgage and home equity loan	Not used		Represented in another variable

B25081_007	Housing units with a mortgage, contract to purchase, or similar debt:!!No second mortgage and no home equity loan	Not used		Represented in another variable
B25081_008	Housing units without a mortgage	Not used		Represented in another variable

Table S38. Goodness-Of-Fit Statistics of Factor Analysis for 1 to 12 Factors

n Factors	Chi Squared	Degrees of Freedom	Fit[1]	Fit (Off)[2]	RMSEA[3]	RMS[4]	Cumulative Variance	Variance Explained
1	4656012	702	0.512	0.55	0.2	0.208	0.233	0.233
2	1294535	664	0.82	0.875	0.17	0.11	0.199	0.415
3	640289	627	0.889	0.938	0.153	0.077	0.153	0.498
4	364600	591	0.921	0.965	0.139	0.058	0.163	0.554
5	185916	556	0.943	0.982	0.124	0.042	0.088	0.601
6	110019	522	0.955	0.989	0.119	0.032	0.058	0.633
7	82662	489	0.961	0.992	0.115	0.028	0.052	0.652
8	55893	457	0.966	0.995	0.112	0.023	0.086	0.672
9	42903	426	0.97	0.996	0.11	0.02	0.122	0.686
10	34313	396	0.972	0.997	0.109	0.018	0.031	0.698
11	26656	367	0.974	0.997	0.104	0.016	0.083	0.711
12	21461	339	0.976	0.998	0.102	0.014	0.033	0.722

[1] $Fit = \frac{\sum r_{ij}^2 - \sum \hat{r}_{ij}^2}{\sum r_{ij}^2}$ estimates how the factor model reproduces the sample correlation matrix, where r_{ij} is the $[i, j]$ element of the sample correlation matrix, and $\hat{r}_{ij}$ is the corresponding element of the model-implied estimate.

[2] $Fit(Off) = \frac{\sum_{i \neq j} r_{ij}^2 - \sum_{i \neq j} \hat{r}_{ij}^2}{\sum_{i \neq j} r_{ij}^2}$ estimates how the factor model reproduces the off-diagonal elements of the sample correlation matrix

[3] The root mean square error of approximation. This statistic is based on normal theory and constructed with the Chi-squared statistic and so was not used for the same reason the Chi-squared statistic was not used in this study: because they are sensitive to large sample sizes.

[4] The root mean square of the residuals. An empirical estimate of RMSEA.

Table S39. Sample Demographic Characteristics

**BRFSS**[1]		**ACS**[2]	
Variable	*Percent*	*Variable*	*Percent ± Percent Margin of Error*
**Sex**			
Male	42.3	Male	49.2 ±0.1
Female	57.7	Female	50.8 ±0.1
**Education**			
No High School Diploma	7.8	Less than 9th grade	5.7 ±0.1
High School Diploma	55.2	9th to 12th grade, no diploma	7.6 ±0.1
College Graduate	36.6	High school graduate (includes equivalency)	27.8 ±0.1
No response	0.4	Some college, no degree	21.1 ±0.1
		Associate's degree	8.1 ±0.1
		Bachelor's degree	18.5 ±0.1
		Graduate or professional degree	11.2 ±0.1
**Race/Ethnicity**			
White only, Non-Hispanic	76.1	White only, Non-Hispanic	62.3 ±0.1
Black only, Non-Hispanic	7.8	Black or African American only, Non-Hispanic	12.3 ±0.1
Other race only, Non-Hispanic	4.5	American Indian and Alaska Native only, Non-Hispanic	0.7 ±0.1
Multiracial, Non-Hispanic	1.8	Asian only, Non-Hispanic	5.1 ±0.1
Hispanic	8.1	Native Hawaiian and Other Pacific Islander only, Non-Hispanic	0.2 ±0.1
Don't know/Not sure/Refused	1.7	Some other race only, Non-Hispanic	0.2 ±0.1
		Two or more races, Non-Hispanic	2.2 ±0.1
		Hispanic or Latino (of any race)	17.1 ±0.1
**Income level (all sources)**			
Less than $10,000	4.2	Less than $10,000	7.2 ±0.1
$10,000 to less than $15,000	4.4	$10,000 to $14,999	5.3 ±0.1
$15,000 to less than $20,000	6.1	$15,000 to $24,999	10.6 ±0.1
$20,000 to less than $25,000	7.3	$25,000 to $34,999	10.1 ±0.1
$25,000 to less than $35,000	8.9	$35,000 to $49,999	13.4 ±0.1
$35,000 to less than $50,000	11.8	$50,000 to $74,999	17.8 ±0.1
$50,000 to less than $75,000	13.2	$75,000 to $99,999	12.1 ±0.1
$75,000 or more	26.1	$100,000 to $149,999	13.1 ±0.1
Don't know/Not sure/Refused/ Missing	18.0	$150,000 to $199,999	5.1 ±0.1
		$200,000 or more	5.3 ±0.1
**Age**			
18 to 24	5.5	Under 5 years	6.3 ±0.1
25 to 29	4.5	5 to 9 years	6.5 ±0.1
30 to 34	5.2	10 to 14 years	6.5 ±0.1
35 to 39	5.6	15 to 19 years	6.7 ±0.1
40 to 44	5.9	20 to 24 years	7.1 ±0.1
45 to 49	6.9	25 to 34 years	13.5 ±0.1
50 to 54	9.0	35 to 44 years	12.8 ±0.1
55 to 59	10.5	45 to 54 years	13.9 ±0.1
60 to 64	11.3	55 to 59 years	6.6 ±0.1
65 to 69	11.2	60 to 64 years	5.8 ±0.1
70 to 74	8.7	65 to 74 years	7.9 ±0.1
75 to 79	6.4	75 to 84 years	4.3 ±0.1
80 or older	8.3	85 years and over	1.9 ±0.1
Don't know/Refused/Missing	1.2		
**Employment Status**			
Employed	48.9	In labor force	63.7 ±0.1
Out of work	4.2	Civilian labor force	63.3 ±0.1
Not in labor force	46.0	Employed	58 ±0.1
Refused	0.9	Unemployed	5.2 ±0.1
		Armed Forces	0.4 ±0.1
		Not in labor force	36.3 ±0.1

[1] Data was acquired from the BRFSS 2015 Codebook Report [3].

[2] Data was acquired from the 2015 ACS 5-Year Estimates Data Profiles. Table IDs DP02, DP03, DP04, and DP05 [4].

References

1. U.S. Census Bureau. American Community Survey (ACS): Table IDs Explained, 2017. Available online: https://www.census.gov/programs-surveys/acs/guidance/which-data-tool/table-ids-explained.html.
2. U.S. Census Bureau. American Community Survey: Table Shells. Available online: https://www2.census.gov/programs-surveys/acs/summary_file/2015/documentation/user_tools/ACS2015_Table_Shells.xlsx.
3. Centers for Disease Control and Prevention. Behavioral Risk Factor Surveillance System 2015 Codebook Report, 2016. Available online: https://www.cdc.gov/brfss/annual_data/2015/pdf/codebook15_llcp.pdf.
4. U.S. Census Bureau. American Community Survey: Data Profiles Report. Available online: https://www.census.gov/acs/www/data/data-tables-and-tools/data-profiles/2015/.
